# Tests of Artificial Neural Network-Based Diabatization Approaches on Simple 1D Models

**DOI:** 10.1021/acs.jctc.5c00083

**Published:** 2025-07-15

**Authors:** Martina Ćosićová, Thierry Leininger, René Kalus

**Affiliations:** † Department of Applied Mathematics, Faculty of Electrical Engineering and Computer Science, VŠB—Technical University of Ostrava, 17. listopadu 2172/15, 708 00 Ostrava−Poruba, Czech Republic; ‡ Université de Toulouse, CNRS UMR5626, Laboratoire de Chimie et Physique Quantiques, 118 Route de Narbonne, 31062 Toulouse Cedex 09, France

## Abstract

Recently, a novel diabatization scheme has been proposed [ ShuY., ; TruhlarD. G., J. Chem. Theory Comput. 2020, 16, 6456–6464]32886513 10.1021/acs.jctc.0c00623 using artificial neural networks. Most importantly, the method almost exclusively requires the knowledge of adiabatic energies, which are routinely obtained from ab initio calculations. However, many questions related to the favorable performance of the method remain unanswered. In the present paper, some of these questions are considered for selected one-dimensional models with one configurational variable. In particular, various activation functions are tested, including nonlinear ones in the output layer, the effect of the regularization term in the loss function is analyzed, and computationally cheap extensions of training sets are proposed. Significant improvements of the performance of the original method have been achieved.

## Graphical abstract

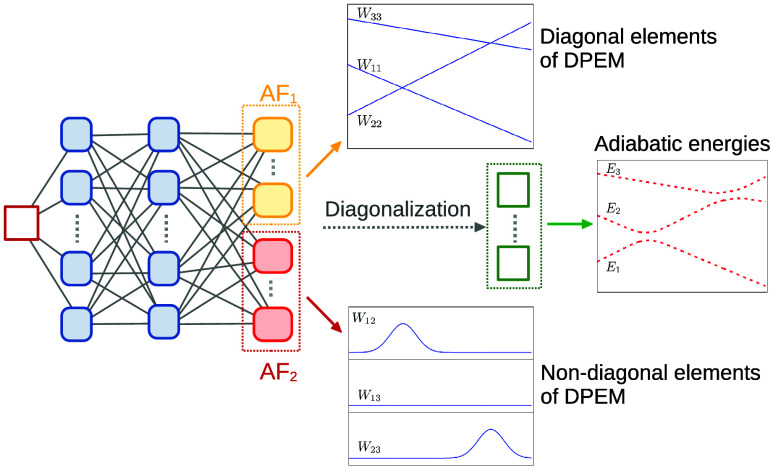

## 1 Introduction

In many molecular processes, including high-energy collisions, electronic postexcitation relaxation and/or dissociation dynamics, etc., the framework of the Born–Oppenheimer approximation and the time evolution running on a single Potential energy surface (PES) is not adequate. Then, several (*N*_s_) electronic states are to be considered simultaneously as well as transitions between them, and a single potential energy surface (PES) must be replaced by an *N*_s_-dimensional electronic potential energy matrix (PEM). See, e.g., ref 1 for a comprehensive introduction.

Usually, the eigenstates of the electronic Hamiltonian, called adiabatic states, are considered. In this case, the PEM is diagonal with adiabatic electronic energies (parametrically dependent on nuclear positions, **R**) on its diagonal and zeros elsewhere. The probabilities of transitions between the electronic states are then given by the first-order and second-order nonadiabatic coupling (NACs), **F**_*jk*_ ≡ ⟨ψ_*j*_ |∇_R_ψ_*k*_⟩ and $G_{\mathrm{jk}}\equiv \frac{1}{2}\left\langle \psi_{j}\right|\Delta_{R}\psi_{K}\rangle$ , respectively, where ψ_*j*_ and ψ_*K*_ are adiabatic electronic wave functions, ∇_R_ denotes the gradient with respect to mass-weighted nuclear coordinates, Δ_R_ is corresponding multidimensional Laplace operator, and brackets represent integration over electronic positions (see, e.g., ref 1 and/or a brief summary given in the Supporting Information Material). The main advantage of such an approach is that the adiabatic energies can be routinely obtained from standard quantum chemistry calculations which are easily performed using optimized quantum chemistry software packages. The NACs are, however, not as easily obtained, which represents one of the most important drawbacks of using the adiabatic representation in calculations. Another disadvantage consists in a nonsmooth dependence of the adiabatic energies on nuclear configurations in crossing regions, e.g., at conical intersections and, even more importantly, in the fact that the NACs diverge in these regions.^2^

Alternatively, a diabatic representation of the PEM (see, e.g., ref 2) can be used. Here, the electronic Hamiltonian is expanded against approximately diabatic electronic states for which the NACs are essentially zero. It is achieved at the expense of having a nondiagonal diabatic potential energy matrix (DPEM), the off-diagonal terms of which represent transition probabilities between different diabatic states. The most important advantage of using the diabatic representation in, e.g., semiclassical dynamical simulations is that DPEMs are free of any divergences and their elements, both diagonal and off-diagonal, are always smooth functions of nuclear coordinates. Unfortunately, the diabatic representation is not as easily and routinely obtained as the adiabatic one. As a consequence, an essential effort has been devoted to constructing diabatic representations for specific systems of physical and/or chemical relevance (see, e.g., references cited in ref 2).

Having in mind the rapid growth of machine learning (ML) methods seen in last decades and their massive proliferation into various domains of science and technology, it is not surprising that numerous studies devoted to the use of ML methods in computational chemistry have recently appeared as well. Specifically artificial neural network (ANNs) have experienced very successful applications in computational chemistry (see, e.g., refs 3,4 , and^5^ for some recent reviews on interactions modelings via ANNs), mainly due to their low computational costs and a high ability to approximate a wide range of functions. In particular, they have been proved as representative and reliable models of PESs.^3,4^ In addition, the ANNs have also been used to approximate DPEMs and/or their parts, often specifically tailored for particular systems. An example of this approach is the diabatization by ansatz.^6,7^

In general, considering the use of ANNs in solving the problem of diabatization, only incomplete results have been obtained so far with most of the studies considering two electronic states only. Individual approaches differ by the quantities used to train the ANNs. The most common quantities include adiabatic energies,^8–10^ diabatic energies obtained either by ab initio calculations^11^ or from a semiempirical model,^12^ and the interstate coupling gradients,^13–15^ to give some typical examples. Note also that recently another machine learning method, kernel ridge regression, has as well been used for solving the diabatization problem.^16^

In our work, we have been inspired by the diabatization by deep neural network (DDNN) approach first published in ref 17. It mainly differs from the previously mentioned methods by its generality and applicability to any number of simultaneously treated electronic states.^18–20^ The method uses a feed-forward neural network with an input layer containing nuclear coordinates, two hidden layers, and two output layers, one representing the DPEM and the other containing adiabatic energies obtained by diagonalizing the DPEM. Training sets then comprise a combination of the elements of DPEM and adiabatic energies, with the diabatic data provided only on the boundary of the studied region of nuclear coordinates where, typically, the diabatic and adiabatic representations are assumed to coincide, while the adiabatic energies are given at a much greater number appropriately distributed throughout the region. In an immediately following work,^21^ the method was further improved by adding another term to the loss function to make the neural network invariant with respect to the permutations of identical particles. In subsequent studies,^19,20^ a parametrically managed postactivation function (PMAF) was introduced to ensure the correct behavior of the approximated diabatic representation in the asymptotic region of the configuration space. However, the two extensions are of only limited use in the case of the presently studied models, the former improvement being not necessary at all and the latter applying to only one of the ”more realistic” models considered in Section 3.4.1 (see also Section 4 of the Supporting Information material).

Although the diabatization using ANNs is a promising approach, many details still remain unresolved. Most importantly, the DDNN method of ref 17 is expected to get a correct diabatic representation if almost exclusively adiabatic data are used for the ANN training, which makes the task fundamentally under-determined. Under such conditions, there are infinitely many solutions of the diabatization problem, which inevitably raises a question about how reliably the method is able to find the correct solution.[Fn fna2] The main goal of this work is to carry out a systematic investigation of the performance of the DDNN method, extended in some directions, on selected simple one-dimensional (1D) models in order to analyze its specific behavior and possible limitations. As a side effect, an optimization of selected adjustable parameters of the method has been performed. As is usual, under a 1D model we mean that both the adiabatic energies and the DPEMs elements depend on a single parameter (nuclear coordinate). Such simplified models have often been used in the literature to test particular diabatization approaches since they are simple enough to avoid technical issues which often occur in many dimensions. As such, the 1D models may thus provide a clearer idea about the performance of tested methods. Importantly, the 1D models can be used to represent real systems like diatomic molecules or even multiatomic molecules in which the internal degrees of freedom are, except for one, frozen due to their stiffness.

As will be shown later in this work, the efficiency of the DDNN method is influenced by many parameters, like the design of a loss function used in ANN training, the number of hidden layers and the number of neurons in them, or by the choice of the activation functions used in hidden and output layers. In this work, we therefore propose certain modifications of the original method, consisting mainly in an appropriate setting of selected internal parameters, which in the case of the tested systems leads to a significantly better performance of the method as compared with its original proposal. Importantly, all the extensions and modifications of the original method of ref 17 we propose in the present work following the analysis we have performed can be directly included in the original method as well as in any of its extensions.^19–21^

In the rest, the present paper is organized as follows. First, a brief account of basic theoretical, methodological, and computational approaches used in our work is given in Section 2. In particular, an overview of the ANN methodology we use is given in Section 2.1 and numerical tests we have performed are specified in Section 2.2. In addition, a short summary of the adiabatic and diabatic representations is provided for reader’s convenience in the Supporting Information Material. Then, the main results are presented and discussed in Section 3, namely, the performance of various activation functions considered in ANNs used to represent DPEMs (Section 3.1) and the role of the regularization term included in the loss function employed in training the ANNs (Section 3.2). In addition, a generalized method for preparing training sets, as discussed in Section 3.3, is described in detail in the Supporting Information material. Finally, conclusive remarks are given in Section 4.

## 2 Theory, Methods, and Computations

In this section, mainly issues related to the representation of DPEM through ANNs are discussed. The DDNN approach of ref 17 is mostly followed though the notation used in our work is somewhat different. For reader’s convenience, a table of correspondence of symbols used here and in ref 17 is provided in the Supporting Information Material (see Section 1 therein). A brief summary of the underlying theory of adiabatic and diabatic representations is for completeness also provided in the Supporting Information Material.

### 2.1 Neural Network Structure and Training

For the approximation of an *N*_S_ × *N*_S_ DPEM, a feed-forward fully connected neural network is used^17^ with one input layer, two output layers, and, in general, *N*_H_ hidden layers. Following ref 17, two hidden layers (*N*_H_ = 2) are used in the present work.

The first output layer (hereafter called the DPEM layer or simply the diabatic layer) contains $N_{D}=\frac{1}{2}N_{S}\left(N_{S}+1\right)$ neurons, *y*_*j*_^D^, that represent individual elements of the DPEM. The second output layer (adiabatic) includes *N*_s_ neurons, *y*_j_^A^, representing the adiabatic energies of the system obtained from the diabatic layer by diagonalizing the corresponding DPEM. The individual hidden layers generally consist of *K*_1_,···,*K*_*N*_H__ hidden neurons, *y*_*j*_^(1)^, ···, *y*_*j*_^(*N*_H_)^. Two hidden layers are used in the present work with *K*_1_ = *K*_2_ = 30, as proposed in ref 17. Finally, the input layer contains a vector of input parameters (coordinates), **x**, the length of which depends on the dimensionality of the particular problem solved. In this work, where 1D models are considered, the input layer consists of a single neuron. To summarize, an example of the overall structure of a neural network with a single input neuron and two hidden layers (as used in the present work) is shown in Figure 1.

**Figure 1:**
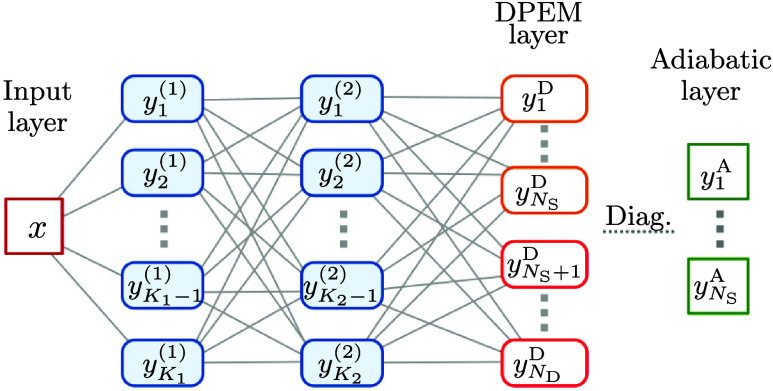
Illustration of a typical neural network structure.

The values of neurons in *m*th hidden layer are given by a standard formula

1 $$y_{k}^{\left(m\right)}=g_{H}\left(z_{k}^{\left(m\right)}\right)$$

where *k* = 1,···,*K*_*m*_, *g*_H_(*z*) is an activation function (AF), and *z*_*k*_^(*m*)^ represents a linear function of the outputs of neurons in (*m* – 1)­th layer

2 $$z_{k}^{\left(m\right)}=b_{k}^{\left(m\right)}+\sum_{j=1}^{K_{m-1}}w_{\mathrm{kj}}^{\left(m\right)}y_{j}^{\left(m-1\right)}$$

Here, *w*_*kj*_^(*m*)^ and *b*_*k*_^(*m*)^ are so-called weights and biases, respectively, and play the role of adjustable parameters. The label attached on the right-hand side of eq 1 to the AF symbol, “H”, is used to distinguish the AF used in hidden layers from that considered in the diabatic layer.

The output values assigned to neurons in the DPEM layer are defined by

3 $$y_{k}^{D}=\{\begin{array}{l} z_{k}^{D},,1\leq k\leq N_{s}\\ g_{D}\left(z_{k}^{D}\right),,N_{s}<k<N_{D} \end{array}$$

with *g*_D_ being an AF used in the DPEM layer to represent the off-diagonal elements of the DPEM while the identity function is used for diagonal elements. In the present work, *g*_D_(*z*) = *z* ^*n*^ where *n* = 1,2,···,5. Let us note that the approach introduced in ref 19, which consists in a multiplication of the DPEM output by a PMAF depending on the internuclear distances in the molecular complex is not used here since its main purpose is to enforce the correct asymptotic behavior of individual DPEM elements and it is not necessary for most of the examples studied in the present work. The only exception is the example discussed in Section 3.4.1 for which the use of the PMAF is considered in the Supporting Information Material (Section 4).

As such, the neural network diabatization approach described above generally follows the method proposed in ref 17. The only major difference is in the form of the AF used in the DPEM layer, *g*_D_. While the identity function, *g*_D_(*z*) = *z*, was used in ref 17 for both the diagonal and off-diagonal terms of the DPEM, power functions of various orders, *g*_D_(*z*) = *z* ^*n*^, are tested for the off-diagonal elements in the present work (see Section 3.1). As shown in Section 3.1, this modification considerably improves the ANN performance. Interestingly, while the use of *g*_D_ for the off-diagonal elements of the DPEM significantly improves the performance of the present approach, the identity AF performs best for the diagonal elements. Its replacement by, e.g., powers of *z* leads to a considerable deterioration of obtained results.

The ANN adjustable parameters, *w*_*kj*_^(*m*)^ and *b*_*k*_^(*m*)^, will be collectively denoted by a vector **Θ** of length *M* (the total number of the ANN adjustable parameters). The search for the optimal values of these adjustable parameters consists in minimizing a loss function, E(Θ) , which is defined as follows

4 $$E\left(\Theta \right)=\frac{1}{N_{\mathrm{TD}}}\sum_{i=1}^{N_{\mathrm{TD}}}{∥y^{D}\left(x_{i}^{D};\Theta \right)-W\left(x_{i}^{D}\right)∥}^{2}+\frac{1}{N_{\mathrm{TA}}}\sum_{i=1}^{N_{\mathrm{TA}}}{∥y^{A}\left(x_{i}^{A};\Theta \right)-E\left(x_{i}^{A}\right)∥}^{2}+\frac{\alpha_{r}}{M}{∥\Theta ∥}^{2}$$

Here, **y** ^D^ and **y** ^A^ are vectors representing the diabatic and adiabatic output layers, respectively, **W** denotes the DPEM, as obtained from the model to be represented by an ANN, expressed in a form of an *N*_d_-dimensional vector (sorted in the same way as **y** ^D^), **E** is an *N*_s_-dimensional vector of eigenvalues of the DPEM corresponding to the given input, and ∥**a**∥ denotes the Eucleidian (*L*_2_) norm of vector **a**. The last term on the right-hand side of eq 4 represents a regularization term^22,23^ and is used to prevent overfitting, α_r_ ≥ 0 is an appropriately adjusted constant. Note that the loss function used in the present work is in principle the same as that of ref 17. Only the value of the regularization parameter was not given in ref 17. and various settings are thus tested in the present work.

Training sets used to optimize the ANNs consist of two parts. One, hereafter called the diabatic training subset, contains *N*_TD_ pairs of {**x**_*i*_^D^,**W**(**x**_*i*_^D^)} representing individual inputs **x**_*i*_^D^ and corresponding DPEM outputs as they follow from a particular model. Note that, in principle, only selected elements of the DPEM can be specified at some input points. The other part of the training set contains *N*_TA_ pairs of {**x**_*i*_^A^,**E**(**x**_*i*_^A^)} representing adiabatic inputs **x**_*i*_^A^ and corresponding adiabatic energies (eigenvalues of **W**), **E**(**x**_*i*_^A^). In the following we will call this part of the training set the adiabatic training subset.

Typically, the adiabatic subset, **x**_*i*_^A^, contains all the points of the diabatic subset, **x**_*i*_^D^, and the number of adiabatic training points is much larger than the number of diabatic ones, *N*_TD_ ≪ *N*_TA_. In an extreme case, considered in ref 17 as well as mostly in the present work (except for Section 3.3) to be directly comparable with ref 17, the diabatic training points may be only given on the boundary of a domain of the configuration space relevant to a specific problem solved. It is particularly useful in situations where adiabatic energies are available in a certain domain of the system configuration space and where the diabatic and adiabatic representations (at least approximately) coincide on its boundary. In 1D systems considered in the present work, this extreme case corresponds to *N*_TD_ = 2 with *x*_1_^D^ and *x*_2_^D^ being the end points of a 1D interval. After an appropriate scaling, the end points may be chosen, e.g., as *x*_1_^D^ = −0.5 and *x*_2_^D^ = +0.5.

### 2.2 Description of Numerical Experiments

For testing the DDNN approach discussed in this study, we have used two artificial 1D models, one taken from ref 17 with two states and an avoided crossing, and the other with three states and two avoided crossings as a straightforward extension of the former model. The two models will be hereafter denoted as $M_{2}$ and $M_{3}$ , respectively, and are graphically illustrated in Figures 2 and 3, respectively. For simplicity, the diagonal elements of both models are linear functions of the configuration parameter, *x*, and can be considered a first-order approximation of more general diabatic potential energy curves near their crossing point. The main goal of the numerical experiments the results of which are presented in this work is to assess the performance of the DDNN approach, i.e., to test the ability of ANNs to correctly represent the two model DPEMs over the whole range of the configuration variable.

**Figure 2:**
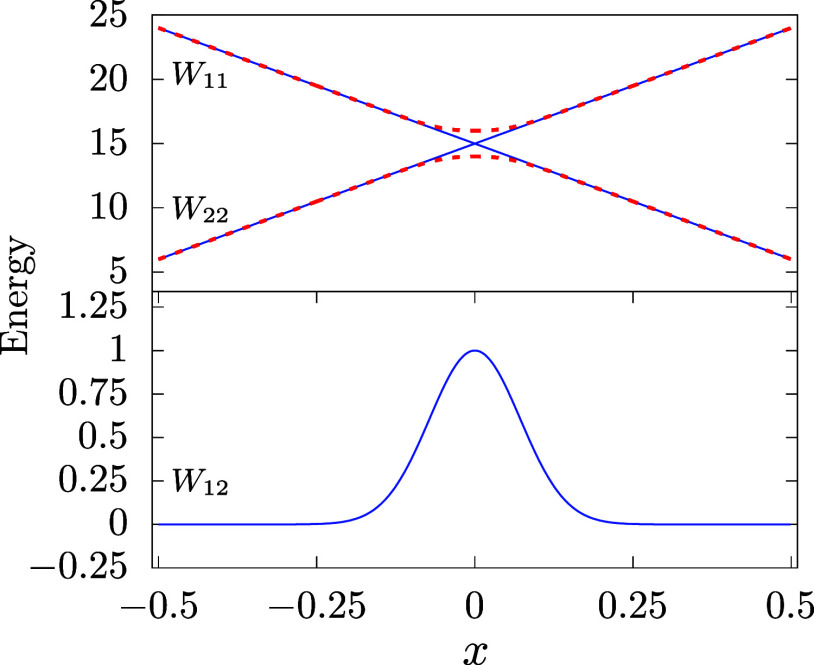
Two-state model DPEM with one avoided crossing as reported in ref 17. Elements of the DPEM are plotted as full lines (blue online), corresponding eigenvalues (adiabatic energies) are shown as (red) dashed lines.

**Figure 3:**
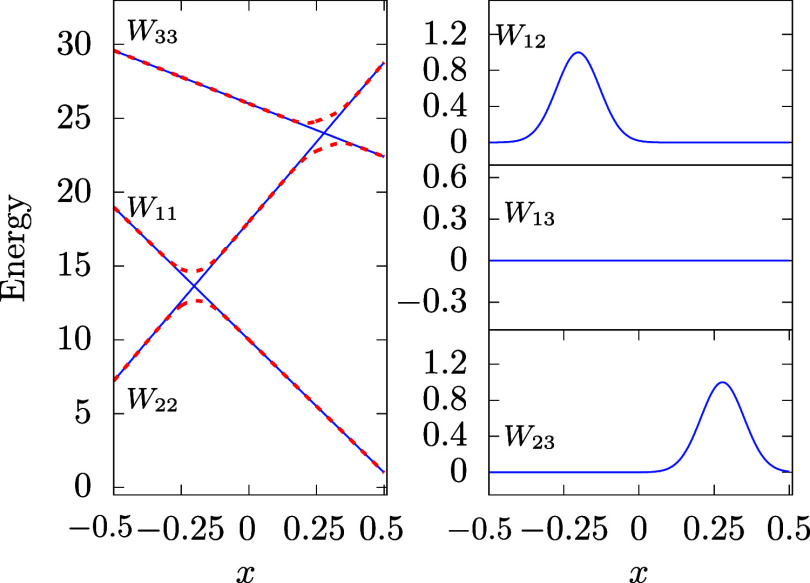
Three-state model of the present study with two avoided crossings. The line styles and colors are the same as in Figure 2.

For training the ANNs through the minimization of the loss function given by eq 4, we have used a Levenberg–Marquardt method^24,25^ with stopping condition being that either the value of the loss function falls below a 10^–7^ tolerance or a maximum number of iterations is reached. In this work, the maximum number of iterations has been set to 150 since after this number is achieved, the value of the loss function does not significantly improve any longer.

As is known, only local minima can be found by local optimization methods (like the Levenberg–Marquardt method) and a particular minimum found in a specific search strongly depends on the initial ansatz used. To get a more comprehensive information, we trained the ANNs repeatedly with different starting points generated randomly using a Glorot method.^26^ Typically, several thousands of repetitions of the optimization procedure have been performed for each particular test calculation (see Section 3). Training sets used to optimize the ANNs consisted of *N*_TA_ = 100 adiabatic training points uniformly distributed over the interval of *x* values, *I* = [−0.5,0.5], with adiabatic energies (eigenvalues of the corresponding DPEM) known in each point and with the DPEM specified as a whole only in two boundary points of the interval. The number of diabatic training points is thus *N*_TD_ = 2.

To quantify the accuracy of results of particular trainings, we have used a mean square deviation between the model DPEM and its ANN representation

5 $$D^{2}=\frac{1}{N}\sum_{k=1}^{N}{∥y^{D}\left(x_{k}\right)-W\left(x_{k}\right)∥}^{2}$$

with *x*_*k*_ being equidistantly distributed over the considered interval of the configuration coordinate and N=1000 . Note also that the signs of the off-diagonal elements of the DPEM, represented by *y*_*i*_^D^(*x*) with *N*_S_ < *i* ≤ *N*_D_, are not uniquely determined. To remove this ambiguity, all combinations of signs of the off-diagonal elements of **y** ^D^ have been considered on the right-hand side of eq 5 and only the best (smallest) value of the right-hand side formula has been taken as $D^{2}$ .

In the present study, we primarily use $D^{2}$ to identify the best solution obtained from repeated training attempts started from various initial guesses with the best solution corresponding to the minimum value of $D^{2}$ found, hereafter denoted as min $\left(D^{2}\right)$ . For real systems, however, for which the DPEM is not known over a sufficiently dense grid of configuration space, finding the best result out of many obtained from different trainings may be a complicated task. It is thus necessary to know what is the performance of DDNN methods ”on the average”. For this reason, we have calculated, in addition to min $\left(D^{2}\right)$ , the median values of $D^{2}$ recorded during a particular series of repeated trainings, med $\left(D^{2}\right)$ , which is supposed to give an idea about a typical range of deviations between the ANN approximation and model DPEM.

Another quantity allowing to assess the performance of DDNN methods is the probability that a result obtained from a single training will deviate from the model DPEM within a given tolerance. For this purpose, we have calculated the mean square deviation of eq 5 specifically for each element of the DPEM, $D_{j}^{2}=\frac{1}{N}\sum_{k=1}^{N}|y_{j}^{D}\left(x_{k}\right)-W_{j}\left(x_{k}\right){|}^{2}$ , and used resulting values to obtain average relative deviations between the model and its ANN approximation, $d_{j}\equiv D_{j}/\Delta_{j}$ , where Δ_*j*_ ≡ max_*k*_{W_*j*_(*x*_*k*_)} – min_*k*_{W_*j*_(*x*_*k*_)} gives a typical range of the *W*_*j*_ values on the tested interval of *x*.[Fn fnb3] Specifically, the probability of all the relative deviations, *d*_*j*_, being below or equal to 1% is used for the two-state model (hereafter we denote this probability by $P_{2}$ ), while a little less strict criterion is adopted in the case of the three-state model (probability $P_{3}$ ) with the accuracy tolerance being 1% for the diagonal elements of the DPEM and 2% for the off-diagonal ones.

## 3 Results and Discussion

### 3.1 Choice of the Activation Function

The particular form of AF is one of the factors that directly influence the performance of ANNs. In our case, there are two different AFs that need to be chosen properly, *g*_H_ for hidden layers and *g*_D_ for the DPEM layer (see eq 3). The main goal of the first series of numerical experiments we have performed has been an investigation of how the efficiency of the DDNN approach is affected if these activation functions are varied.

For testing purposes, we have considered a set of DDNN methods using different activation functions which are summarized in Table 1. In addition to several AFs commonly used in hidden layers (sigmoid, hyperbolic tangent, and GELU;^27^ column *g*_H_), we have also varied the AF in the DPEM layer (column *g*_D_). Specifically, higher powers of the input signal have been considered for the latter, in addition to the standardly employed (first-order) identity function. Note that the DDNN_S_^1^ method is identical to one of the two methods considered in ref 17 while DDNN_G_^1^ is similar to the other method of that reference, which only used the rectifier linear unit (ReLU) as the activation function in hidden layers instead of a smoother GELU function employed in the present work (as proposed, e.g., in ref 19). Since the results obtained for the DDNN_S_^1^ and DDNN_G_^1^ methods represent calculations which are as close as possible to the calculations performed in ref 17 (and to which we compare), they are highlighted in italics in the tables presented below.

**Table 1:** Summary of DDNN Implementations with Different AFs Used in Hidden Layers (*g*_H_) and the DPEM Layer (*g*_D_) as Tested in the Present Work

acronym	*g*_H_	*g*_D_
DDNN_G_^*n*^	$\frac{z}{2}\cdot \left(1+\mathrm{erf}\left(\frac{z}{\sqrt{2}}\right)\right)$	*z* ^*n*^
DDNN_T_^*n*^	tanh(*z*)	*z* ^*n*^
DDNN_S_^*n*^	$\frac{1}{1+e^{-z}}$	*z* ^*n*^

#### Model $M_{2}$

3.1.1

For model $M_{2}$ , we have performed a total of 4736 independent trainings for each of the methods given in Table 1 started from different initial guesses of the weights and biases generated by the Glorot method. In all the trainings, α_r_ = 10^–8^ has been used in the regularization term of the loss function. The results are summarized in Table 2 and Figure 4. In Table 2, the values of the median and minimum of the mean-square deviation between two-state model $M_{2}$ and corresponding ANNs, $D^{2}$ , are reported together with probabilities $P_{2}$ extracted from recorded $D^{2}$ histograms. In Figure 4, cumulative distribution functions are given for $D^{2}$ to provide a more detailed view.

**Figure 4:**
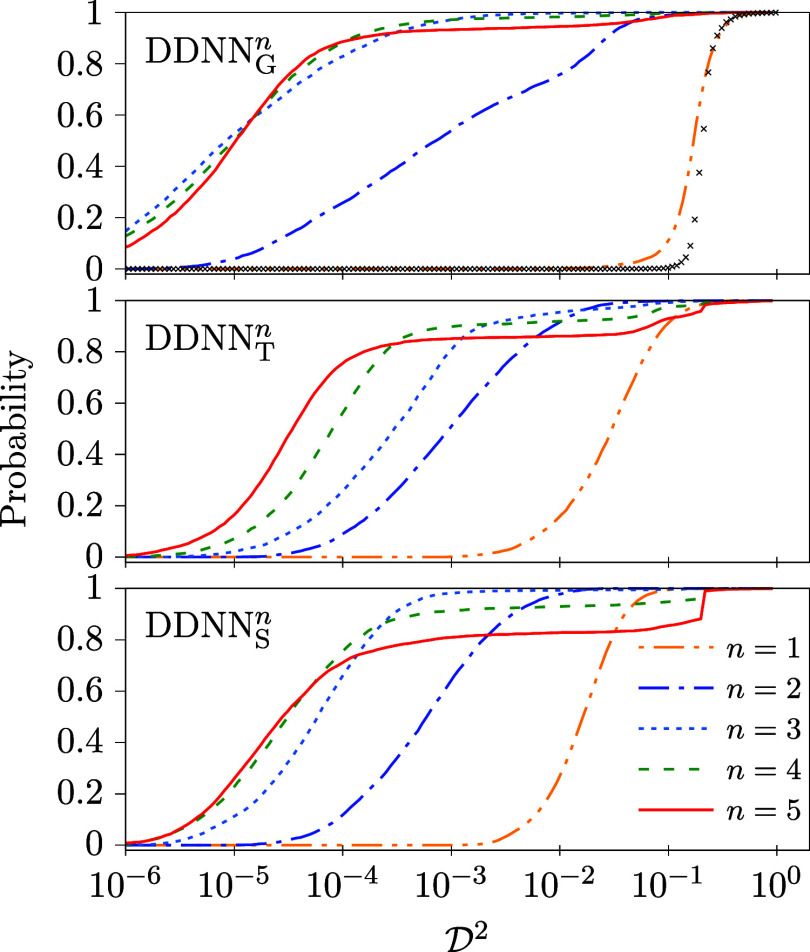
Cumulative distribution functions of $D^{2}$ calculated for model $M_{2}$ and DDNN_*X*_^*n*^ (X = G,T,S, and *n* = 1–5; see Table 1). For comparison, a distribution function which corresponds to the setting of DDNN method adopted in ref 17, i.e., with a linear activation function used in the DPEM layer and α_r_ = 10^–4^, is added in the upper panel (black crosses). For this setting, med $\left(D^{2}\right)=0.21$ and min $\left(D^{2}\right)=1.19\cdot 10^{-2}$ has been obtained (cf. the DDNN_G_^*n*^ column of Table 2).

**Table 2:** Median and Minimum Values of $D^{2}$ , Med­( $D^{2}$ ) and Min­( $D^{2}$ ), Respectively, and Probabilities $P_{2}$ as Obtained for Model $M_{2}$ Using Different Activation Functions Given in Table 1 ^a^

*n*	DDNN_G_^*n*^	DDNN_T_^*n*^	DDNN_S_^*n*^
med $\left(D^{2}\right)$			
1	*0.17*	3.03·10^–2^	*1.63 ·10^–2^*
2	7.23·10^–4^	9.64·10^–4^	5.91·10^–4^
3	8.59·10^–6^	2.98·10^–4^	5.86·10^–5^
4	9.84·10^–6^	8.04·10^–5^	3.13·10^–5^
5	1.03·10^–5^	3.44·10^–5^	2.76·10^–5^
min $\left(D^{2}\right)$			
1	*2.18·10^–3^*	8.04·10^–4^	*1.00·10^–3^*
2	6.58·10^–7^	7.34·10^–6^	9.44·10^–6^
3	4.63·10^–8^	1.56·10^–6^	8.86·10^–7^
4	4.57·10^–8^	6.76·10^–7^	4.40·10^–7^
5	4.19·10^–8^	4.34·10^–7^	3.24·10^–7^
$P_{2}$			
1	*0.00*	0.00	*0.00*
2	2.67·10^–1^	1.13·10^–1^	1.33·10^–1^
3	8.44·10^–1^	3.07·10^–1^	7.16·10^–1^
4	8.93·10^–1^	6.31·10^–1^	7.95·10^–1^
5	8.93·10^–1^	7.83·10^–1^	7.34·10^–1^

a The values of *n* = 1,···,5 correspond to the powers used in the *g*_D_ activation function, see eq 3. All the data have been obtained from 4,736 independent ANN trainings. The values highlighted in italics correspond (DDNN_S_^1^) or are similar (DDNN_G_^1^) to the setting *n* = 1 used in ref 17.

Several conclusions are clear for model $M_{2}$ from both the table and the figure. First and foremost, it is seen at a first glance that the results depend rather strongly on the AFs used, particularly, on the AF in the DPEM layer, *g*_D_. It is nicely illustrated by the values of the $P_{2}$ probability (the last section of Table 2) which represents the chance that the result of an individual ANN training will fall within a preset accuracy tolerance (1% for each element of the DPEM for model $M_{2}$ , see Section 2.2). The values of $P_{2}$ we have got seem to indicate that the standard identity activation function is by far not the best choice for the DPEM layer. Any other *g*_D_ considered in this work outperforms it significantly. In addition, the $P_{2}$ probability rises rapidly if the power is increased in *g*_D_(*z*) = *z* ^*n*^ and reaches a surprisingly high value of about 70 to 90% for *z* = 5. It means that one has exactly such a high chance to get a very accurate ANN representation of the model $M_{2}$ DPEM even for a single ANN training started from a randomly chosen ansatz. Second, the dependence of the accuracy of the ANN representation of model $M_{2}$ on the AF used in hidden layers is much more balanced, though the DDNN_G_^*n*^ methods (using the GELU AF) exhibits a slightly better performance than the other two hidden-layer AFs used.

A more detailed picture can be obtained from Figure 4 where cumulative distribution functions of $D^{2}$ are plotted for the various DDNN methods considered in this work. Like in Table 2, a rather poor performance is seen for the identity AF used in the DPEM layer, *n* = 1, which corresponds to the original choice used in ref 17. Typically, by several orders of magnitude larger $D^{2}$ are obtained in this case as compared to other AFs, *n* ≥ 2. It is also noteworthy that all the distribution functions shown in Figure 4 result from a more or less random search in the ANN weight-bias space. Since the correct solutions (representing the tested model with a full precision) are expected to fill a manifold in this space of a considerably smaller dimension than the space itself, distribution functions with a well pronounced threshold (like those obtained for *n* = 1) are expected to arise from such essentially random trainings. It is simply because a close vicinity of such low-dimensional manifolds will have negligibly small volumes. If the threshold is, on the other hand, shifted to $D^{2}\approx 0$ (like for *n* ≥ 3), one might speculate that a bias toward the correct solutions is in some way inherent in corresponding DDNN methods.

#### Model $M_{3}$

3.1.2

To verify the conclusions learnt for two-state model $M_{2}$ , we have performed a similar series of numerical experiments for the three-state model, $M_{3}$ , under the same conditions with only the number of trainings reduced to 3072. The results are summarized in Table 3 and Figure 5.

**Figure 5:**
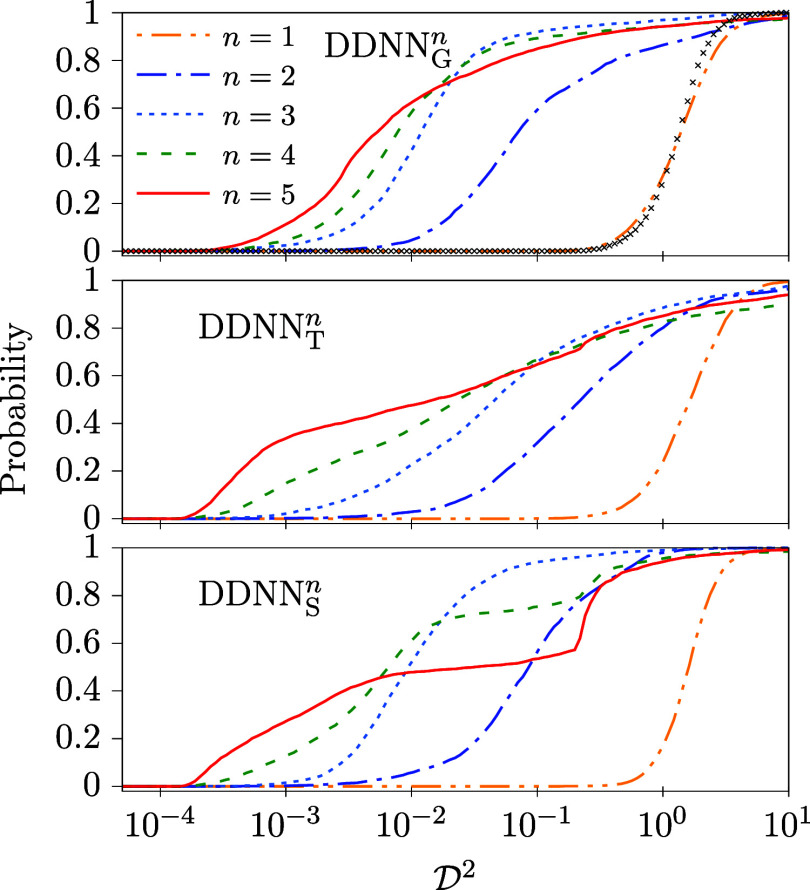
Same as in Figure 4 but calculated for model $M_{3}$ . Like in Figure 4, the black crosses correspond to the original setting of DDNN method of ref 17 (a linear activation function in the DPEM layer and α_r_ = 10^–4^); med $\left(D^{2}\right)=1.25$ and min $\left(D^{2}\right)=8.65\cdot 10^{-2}$ in this case (cf. the DDNN_G_^*n*^ column of Table 3).

**Table 3:** Same as in Table 2 but Calculated for Model $M_{3}$ Using 3072 Independent ANN Trainings

*n*	DDNN_G_^*n*^	DDNN_T_^*n*^	DDNN_S_^*n*^
med $\left(D^{2}\right)$			
1	1.38	1.65	1.60
2	7.03·10^–2^	0.22	8.55·10^–2^
3	1.19·10^–2^	4.63·10^–2^	9.39·10^–3^
4	7.73·10^–3^	2.35·10^–2^	6.58·10^–3^
5	5.35·10^–3^	1.50·10^–2^	3.08·10^–2^
min $\left(D^{2}\right)$			
1	9.42·10^–2^	8.18·10^–2^	0.15
2	8.05·10^–4^	4.03·10^–4^	4.24·10^–4^
3	1.04·10^–4^	1.29·10^–4^	1.89·10^–4^
4	9.48·10^–5^	1.34·10^–4^	1.51·10^–4^
5	1.74·10^–4^	1.33·10^–4^	1.45·10^–4^
$P_{3}$			
1	0.00	0.00	0.00
2	3.26·10^–4^	1.30·10^–3^	1.30·10^–3^
3	1.37·10^–2^	1.33·10^–2^	6.83·10^–3^
4	2.12·10^–2^	1.15·10^–1^	7.94·10^–2^
5	5.79·10^–2^	3.07·10^–1^	2.06·10^–1^

In Table 3, similar data as those given in Table 2 for model $M_{2}$ are summarized for model $M_{3}$ , computed medians and minima of $D^{2}$ and probabilities of getting an ANN optimized within a given tolerance in an individual training event ( $P_{3}$ , see Section 2.2). A first observation that is clear from Table 3 is that all the methods considered perform considerably worse for model $M_{3}$ than for the simpler model $M_{2}$ . Typically, both med $\left(D^{2}\right)$ and min $\left(D^{2}\right)$ are by at least 2 orders of magnitude larger for $M_{3}$ . This difference may be partly attributed to the fact that the $M_{3}$ DPEM contains twice more elements than the DPEM of model $M_{2}$ . Since $D^{2}$ does not take this difference into account, the values of med $\left(D^{2}\right)$ and min $\left(D^{2}\right)$ are expected to be twice as larger for $M_{3}$ even if the DDNN methods performed equally well for both models. However, this factor cannot account for the differences actually seen between the two models in our calculations.

This is further supported by the probability of getting a solution within a given tolerance, $P_{3}$ , which is, despite the fact that a less strict accuracy criterion has been considered for model $M_{3}$ , considerably smaller than corresponding $P_{2}$ probabilities obtained for model $M_{2}$ . For example, even for the most favorable case, DDNN_T_^5^, the $P_{3}$ probability is about 30%, much smaller as compared to the almost 90% probability we have got as the best result for model $M_{2}$ . Moreover, the $P_{3}$ probability typically amounts to only about several percent or even less, again much smaller than what we get for model $M_{2}$ (typically, above 60 to 70%).

Cumulative distribution functions of $D^{2}$ , as obtained for model $M_{3}$ , are plotted in Figure 5. If they are compared with the distribution functions calculated for model $M_{2}$ (Figure 4), one can clearly see that typically much larger values of $D^{2}$ result for model $M_{3}$ . In particular, extremely large values of $D^{2}$ are obtained for DDNN_X_^1^ (X = G,L,S). It means that in many cases the DDNN methods using the identity AF in the DPEM layer have not been able to find a proper approximation of the $M_{3}$ model at all. Clearly, high powers of the input signal are to be used in the DPEM layer activation functions, *g*_D_ = *z* ^*n*^, ideally, *n* = 4 or *n* = 5, to get a sufficiently accurate representation of model $M_{3}$ .

### 3.2 Effect of Regularization

The regularization term in the loss function, eq 4, is primarily used to avoid overfitting, and in the context of the present approach, it is expected^2^ to help ensure smoothness of ANN representations of DPEMs. The regularization term itself, on the other hand, deteriorates the accuracy of an optimal ANN solution and, consequently, putting too much emphasis on it may be contradictory. As a consequence, a trade-off between two principal requirements, the accuracy of a particular ANN representation and its smoothness, has to be found.

In the numerical experiments presented in this section, a series of ANN trainings have been performed for different values of the weighting parameter in the regularization term, α_r_. In these test calculations, the DDNN_X_^5^ (X = G,T,S) methods have been considered since they have mostly lead to the best results presented in the preceding subsection. Like before, resulting data are reported in Tables 4 and 5, and Figures 6 and 7.

**Figure 6:**
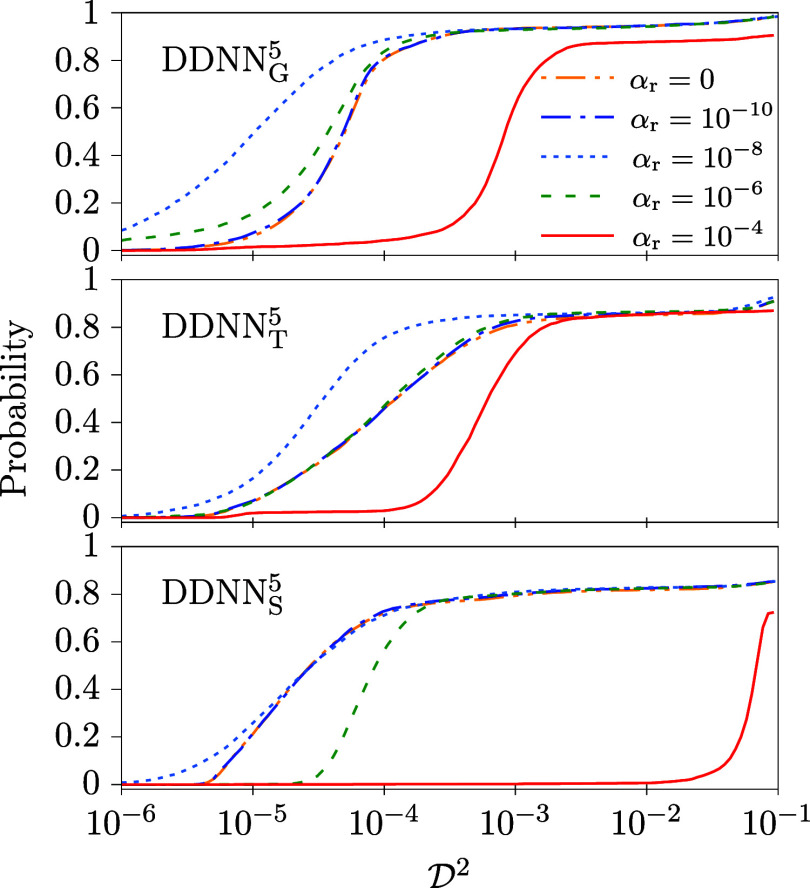
Cumulative distribution functions of $D^{2}$ as obtained for model $M_{2}$ , DDNN_*X*_^5^ (X = G,T,S), the off-diagonal DPEM activation function, eq 3, *g*_D_(*z*) = *z* ^5^, and for different values of α_r_ in the regularization term of eq 4. Note that the α_r_ = 0 curves almost coincide with the curves corresponding to α_r_ = 10^–10^.

**Figure 7:**
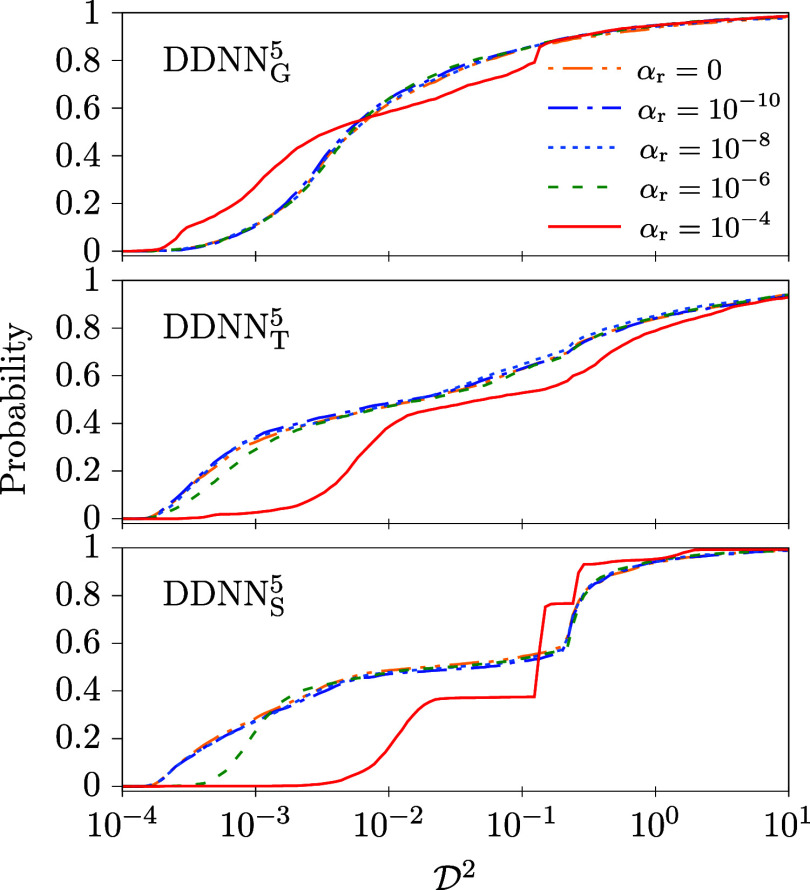
Same as in Figure 6 but calculated for model $M_{3}$ . Like in Figure 6 (model $M_{2}$ ), the α_r_ = 0 and α_r_ = 10^–10^ curves are more or less identical and, unlike model $M_{2}$ , they coincide with the curves obtained for α_r_ = 10^–8^.

**Table 4:** Median and Minimum Values of $D^{2}$ , Med $\left(D^{2}\right)$ and Min $\left(D^{2}\right)$ , Respectively, and Probabilities $P_{2}$ as Obtained for Model $M_{2}$ Using Different Values of α_r_ in the Regularization Term of Equation 4 ^a^

α_r_	DDNN_G_^5^	DDNN_T_^5^	DDNN_S_^5^
med $\left(D^{2}\right)$			
0	5.01·10^–5^	1.22·10^–4^	2.73·10^–5^
10^–10^	4.87·10^–5^	1.20 ·10^–4^	2.81·10^–5^
10^–8^	1.03·10^–5^	3.44·10^–5^	2.76·10^–5^
10^–6^	3.75·10^–5^	1.14·10^–4^	8.58·10^–5^
10^–4^	8.34·10^–4^	6.17·10^–4^	6.88·10^–2^
min $\left(D^{2}\right)$			
0	2.78·10^–7^	1.64·10^–6^	3.20·10^–6^
10^–10^	3.56·10^–7^	1.16·10^–6^	2.76·10^–6^
10^–8^	4.19·10^–8^	4.34·10^–7^	3.24·10^–7^
10^–6^	3.73·10^–8^	5.57·10^–7^	2.58·10^–6^
10^–4^	8.28·10^–7^	3.92·10^–6^	5.93·10^–6^
$P_{2}$			
0	8.24 ·10^–1^	5.20·10^–1^	7.37·10^–1^
10^–10^	8.29·10^–1^	5.17·10^–1^	7.43·10^–1^
10^–8^	8.93·10^–1^	7.83·10^–1^	7.34·10^–1^
10^–6^	8.55·10^–1^	5.33·10^–1^	6.47·10^–1^
10^–4^	5.72·10^–2^	3.99·10^–2^	1.68·10^–3^

a All the data have been obtained from 4,736 independent ANN trainings with the off-diagonal DPEM activation function, eq 3, being *g*_D_(*z*) = *z* ^5^.

**Table 5:** Same as in Table 4 But Calculated for Model $M_{3}$ Using 3072 Independent ANN Trainings

α_r_	DDNN_G_^5^	DDNN_T_^5^	DDNN_S_^5^
med $\left(D^{2}\right)$			
0	5.37·10^–3^	1.78·10^–2^	1.89·10^–2^
10^–10^	4.80·10^–3^	1.40·10^–2^	5.26·10^–2^
10^–8^	5.35·10^–3^	1.50·10^–2^	3.08·10^–2^
10^–6^	5.17·10^–3^	1.87·10^–2^	2.67·10^–2^
10^–4^	3.50·10^–3^	5.13·10^–2^	1.32·10^–1^
min $\left(D^{2}\right)$			
0	1.31·10^–4^	1.37·10^–4^	1.44·10^–4^
10^–10^	1.29·10^–4^	1.31·10^–4^	1.43·10^–4^
10^–8^	1.74·10^–4^	1.33·10^–4^	1.45·10^–4^
10^–6^	1.38·10^–4^	1.38·10^–4^	1.55·10^–4^
10^–4^	1.20·10^–4^	2.72·10^–4^	2.00·10^–4^
$P_{3}$			
0	5.34·10^–2^	2.70·10^–1^	2.20·10^–1^
10^–10^	5.24·10^–2^	2.96·10^–1^	2.06·10^–1^
10^–8^	5.79·10^–2^	3.07·10^–1^	2.06·10^–1^
10^–6^	5.76·10^–2^	2.24·10^–1^	1.00·10^–1^
10^–4^	1.93·10^–1^	2.21·10^–2^	9.76·10^–4^

#### Model $M_{2}$

3.2.1

In the case of two-state model $M_{2}$ , all the test calculations consist, like before, of 4736 trainings with initial values of ANN weights and biases generated using the Glorot method. The results are summarized in Table 4 and Figure 6.

As is clear from Table 4, α_r_ = 10^–8^ leads in general to the best performance, both med $\left(D^{2}\right)$ and/or min $\left(D^{2}\right)$ show mostly a well pronounced minima at exactly this value and the $P_{2}$ probability is maximal for α_r_ = 10^–8^ for two of the three considered DDNN methods. This value of α_r_ represents thus the wanted trade-off between the accuracy and smoothness of the optimized ANNs. It is therefore used throughout this work. Note also that ”too large” values of α_r_ (represented here by α_r_ = 10^–4^) seem to deteriorate outputs of all the considered DDNN methods, in particular the $P_{2}$ probability.

Another interesting feature which is seen in Table 4 is that if the regularization term is completely omitted in eq 4 (α_r_ = 0), the performance of the DDNN methods is in principle not worsen too much. It means that, at least for model $M_{2}$ , the role of the regularization term may not be crucial (see also a similar conclusion for model $M_{3}$ ).

Not surprisingly, the conclusions derived from Table 4 are fully confirmed by the recorded $D^{2}$ distribution functions shown in Figure 6 where the best performance of α_r_ = 10^–8^ is even more clearly articulated.

In ref 17, α_r_ = 10^–4^ was used.^28^ As is clear from Table 4 and Figure 7, such a setting mostly leads to an inferior behavior of the tested methods as compared to other values of α_r_. Note, however, that the fifth power, *n* = 5, has been used in the DPEM activation function here, in contrast to ref 17 where *n* = 1.

#### Model $M_{3}$

3.2.2

Numerical tests performed for model $M_{3}$ comprise 3072 independent trainings each. Resulted data are summarized in Table 5 and Figure 7.

Interestingly and unlike model $M_{2}$ , neither med $\left(D^{2}\right)$ and min $\left(D^{2}\right)$ nor probability $P_{3}$ (see Table 5) any longer show a significant dependence on α_r_ (maybe except for α_r_ = 10^–4^). We thus believe that it is possible to use any value of α_r_ ≤ 10^–6^ without significantly changing the accuracy of the considered DDNN methods, including the α_r_ case. Like for model $M_{2}$ , such an observation may to some extent contradict a hypothesis^2^ that the regularization term is needed to get a smooth behavior of DPEMs represented by ANNs.

These observations are confirmed by the data depicted in Figure 7 where only minor differences can be seen between different distribution functions obtained for α_r_ ≤ 10^–6^ while a major deviation emerges for α_r_ = 10^–4^. Interestingly, (a) for the DDNN_G_^5^ method α_r_ = 10^–4^ leads to the most favorable behavior, unlike the other two methods for which α_r_ = 10^–4^ shows an inferior performance, and (b) the α_r_ = 10^–6^ distribution function deviates visibly from those of α_r_ ≤ 10^–8^ for DDNN_S_^5^. Only α_r_ ≤ 10^–8^ seem to lead to a good accuracy in this case.

### 3.3 Extended Diabatic Training Subsets

Up to now, we have used a minimal training set consisting of only *N*_TD_ = 2 diabatic training points (located at the boundary of the interval of the *x* parameter), as proposed in ref 17, and *N*_TA_ = 100 adiabatic training points (homogeneously distributed over the *x* interval). Hereafter, we will denote this training set as TS_1_. In the previous two subsections, we have verified that even with this reduced training set providing only a very limited information about the DPEM, the DDNN methods discussed in this work are able to accurately reproduce model DPEMs with a rather high probability. For example, for the DNN_G_^5^ method, we have found that the probability of finding a reasonably accurate representation of the two-state model $M_{2}$ is surprisingly high, about $P_{2}\approx 90\%$ . However, after comparing this value with a similar probability calculated for the three-state model $M_{3}$ , $P_{3}\approx 6\%$ , we can clearly see that even though the accuracy requirements used for model $M_{3}$ are a bit less strict than those adopted for model $M_{2}$ , the chance of getting a close-to-correct solution is significantly decreased for model $M_{3}$ .

This may seem rather discouraging since a main goal of the development of ANN diabatization methods is their application to realistic, i.e., more complex models often comprising multiple states with numerous state interactions. Following the observation made above, it can be expected that the efficiency of the methods discussed in this work will be significantly decreased if the methods are applied to such more involved problems.

A possible solution to overcome this issue is to enhance the diabatic part of training sets. A straightforward option for this may be to generate additional diabatic training points using ab initio calculations. However, it is usually a highly involved task, and it holds even if ready-to-use software solutions are employed. In this work, we propose a different way for generating additional diabatic training points which consists in propagating the points given at the boundary of a domain of configurational parameters inside the domain. In this way, the proposed algorithm stems from the original method of ref 17 and straightforwardly extends it.

Specifically, we propose an algorithm applicable to 1D systems discussed in this work. The algorithm may, however, be used to improve the behavior of DDNN approaches in the case of multidimensional systems as well since most of training strategies usually adopt an iterative scheme using 1D cuts through the multidimensional hypersurfaces as much as accurately represented as a first step. Hereafter we will refer to this algorithm as to the diabatic training set generating (DTSG) algorithm. The algorithm is based on the following two assumptions (see the Supporting Information Material for details)

- Adiabatic and diabatic representations are (approximately) equal outside avoided crossing regions
- The diabatic energies (diagonal terms of the DPEM) cross in/around minima of functions *d*_*j*_(*x*) = *E*_*j*+1_(*x*) – *E*_*j*_(*x*), where *E*_*j*+1_ and *E*_*j*_ stand for the (*j* + 1)-th and *j*-th adiabatic energy, respectively.[Fn fnd4]

The “outside of an avoided crossing” is defined by two radii, *r*_1_ and *r*_2_, with the former used for the diagonal elements of the DPEM and the latter for the off-diagonal ones. A diagonal element of the DPEM corresponding to training point *x* is then assumed to be outside the region of an avoided crossing located at *x*_0_ if *x* ∉ [*x*_0_ – *r*_1_, *x*_0_ + *r*_1_] and an off-diagonal one if *x* ∉ [*x*_0_ – *r*_2_, *x*_0_ + *r*_2_]. Parameters *r*_1_ and *r*_2_ are expected to be “manually” adjusted and specify a particular variant of the DTSG algorithm.

In the present work, we have specifically applied the DTSG algorithm to model $M_{3}$ for which the DDNN methods have led to an inferior efficiency if used together with the original training set TS_1_. Using the DTSG algorithm, we have created two more training sets. One with *r*_1_ = *r*_2_ = 1.0, which means that *W*_13_ is set to zero in all the training points while the remaining *W*_*ij*_ are specified at the boundary of the *x* interval, like in the original training set TS_1_. We will denote this enhanced training set as TS_2_. The other training set, TS_3_, has been obtained for *r*_1_ = 0.15 and *r*_2_ = 0.3. The diabatic training points added to the training set are graphically represented in Figure 8. An important point which should be emphasized is that out of the crossing regions, the diagonal elements of the DPEM are set to the values of corresponding adiabatic energies and the off-diagonal ones are set to zero. So, no additional calculations are needed.

**Figure 8:**
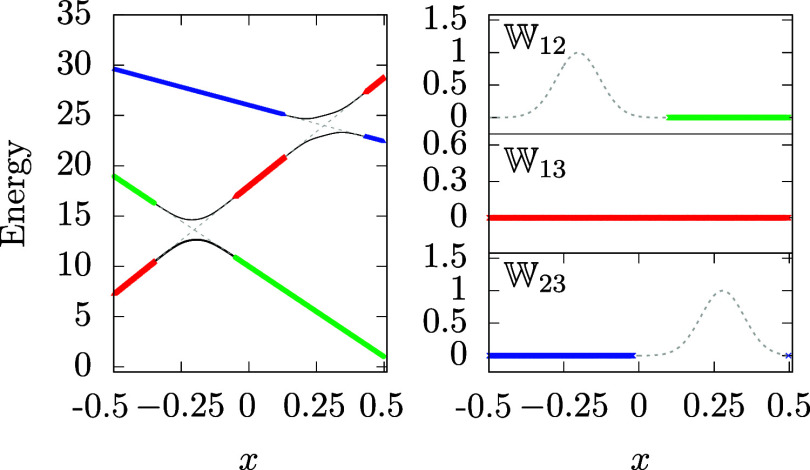
Graphical representation of the TS_3_ training set generated for model $M_{3}$ using the DTSG algorithm with *r*_1_ = 0.15 and *r*_2_ = 0.3. Solid curves represent the adiabatic energies of the model, dashed lines are used to depict the DPEM elements, and colored dots are used for the diabatic training points added by the DTSG algorithm as described in the main text.

The results we have obtained for model $M_{3}$ for the extended training sets, TS_2_ and TS_3_, are compared with outputs of trainings performed for the original training set, TS_1_, in Table 6 and Figure 9.

**Figure 9:**
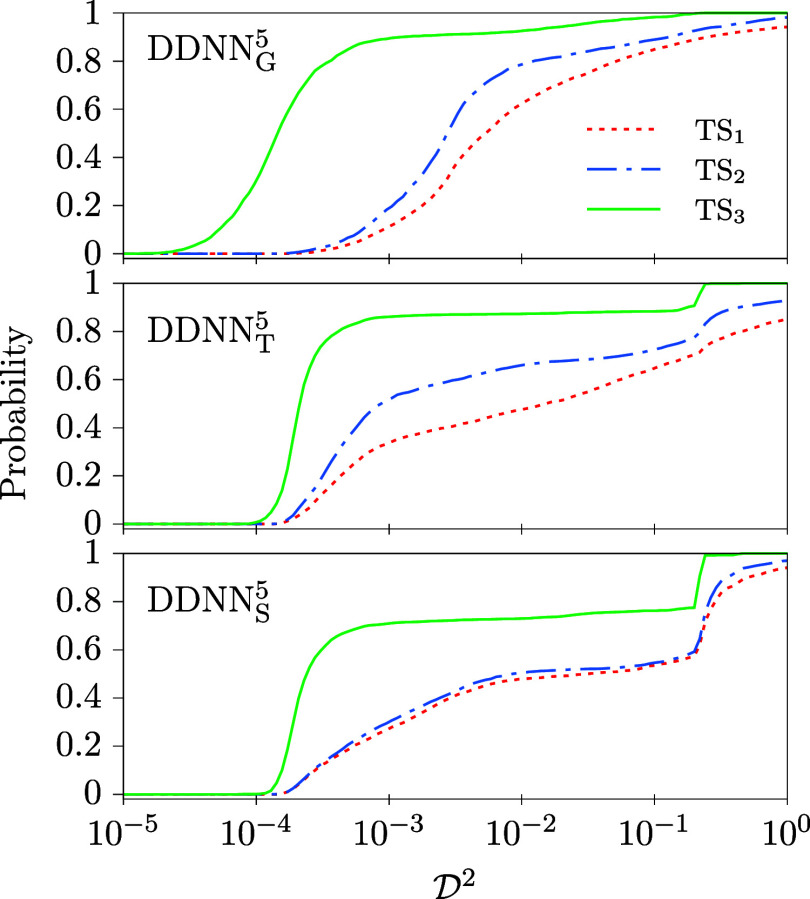
Cumulative distribution functions of $D^{2}$ calculated for model $M_{3}$ , DDNN_X_^5^ (X = G,T,S), and different training sets considered in Table 6. Note that a different scale is used on the horizontal axis than in the other figures reported for model $M_{3}$ so that the improvement obtained for TS_3_ and DDNN_G_^5^ (the upmost panel) is seen in its full extent.

**Table 6:** Median and Minimum Values of $D^{2}$ , Med $\left(D^{2}\right)$ and Min $\left(D^{2}\right)$ , Respectively, and Probabilities $P_{3}$ as Obtained for Model $M_{3}$ Using the Original Training Set (TS_1_) and Two Extended Sets with Enhanced Diabatic Training Points (TS_2_ and TS_3_)­^a^

Training set	DDNN_G_^5^	DDNN_T_^5^	DDNN_S_^5^
med $\left(D^{2}\right)$			
TS_1_	5.35·10^–3^	1.50·10^–2^	3.08·10^–2^
TS_2_	2.73·10^–3^	8.74·10^–4^	8.43·10^–3^
TS_3_	1.45·10^–4^	2.12·10^–4^	2.37·10^–4^
min $\left(D^{2}\right)$			
TS_1_	1.74·10^–4^	1.33·10^–4^	1.45·10^–4^
TS_2_	1.30·10^–4^	1.36·10^–4^	1.34·10^–4^
TS_3_	1.28·10^–5^	7.53·10^–5^	7.39·10^–5^
$P_{3}$			
TS_1_	5.79·10^–2^	3.07·10^–1^	2.06·10^–1^
TS_2_	1.07·10^–1^	4.66·10^–1^	2.25·10^–1^
TS_3_	8.68·10^–1^	8.46·10^–1^	6.97·10^–1^

a Like before, the presented data have been obtained, for each training set, from 3,072 independent ANN trainings with the off-diagonal DPEM activation function, eq 3, being *g*_D_(*z*) = *z* ^5^; the TS_1_ data have been taken from Table 3.

Like before, the table provides the median and minimum values of $D^{2}$ , min $\left(D^{2}\right)$ and med $\left(D^{2}\right)$ , respectively, the and probabilities of achieving a desired accuracy, $P_{3}$ . All the three quantities show that the use of the second training set, TS_2_, does not lead to a significant improvement of the behavior of any of the considered diabatization methods as compared with TS_1_. This can, however, be expected since the two training sets, TS_1_ and TS_2_, differ by training points corresponding to only one element of the DPEM, namely W_13_. After employing TS_3_, on the other hand, the picture is quite different. In this latter case, there is a significant improvement in all the quantities we consider, which are mostly improved by an order of magnitude or even more. It is quite encouraging since, as mentioned above, the values of DPEM added to TS_3_ are only approximate and do not require any additional calculation.

The picture seen in Table 6 is further confirmed by Figure 9 where cumulative distributions of $D^{2}$ are provided as obtained for the three training sets. While training set TS_2_ usually leads to similar curves as TS_1_, the TS_3_ training set yields $D^{2}$ distributions significantly shifted to zero. It means that mostly highly accurate solutions are obtained for TS_3_. It is clearly reflected in the last section of Table 6 where the $P_{3}$ probability (the chance of finding an accurate solution) is considerably enhanced for TS_3_.

### 3.4 Examples of More Realistic Models

This last part of Section 3 is devoted to testing the DDNN_G_^*n*^ methods on two additional, more realistic 1D examples represented by different cuts of thiophenol potential energy surfaces as considered in the original paper.^17^ The adiabatic energies and the DPEM have been obtained from the diabatization model proposed in ref 29 and calculated using subroutines provided in its Supporting Information. Like above, two hidden layers with 30 neurons and the GELU activation function have been used in all these additional calculations. The weight of the regularization term in the loss function has been set to α_r_ = 10^–8^ with α_r_ = 10^–4^ also considered for *n* = 1 to directly compare with ref 17.

#### 3.4.1 Thiophenol: Example 1

As the first example, we have tested the performance of the DDNN_G_^*n*^ methods on a 1D cut of the thiophenol potential energy along the S–H bond length, *R*_S–H_, with the C–C–S–H torsional angle fixed at 0 degrees and all the remaining atoms kept at their ground-state equilibrium positions (particular coordinates have been taken from the Supporting Information to ref 29). In the following numerical experiments, we consider *R*_S–H_ in the range of 0.9 Å to 5.0 Å, as used in Example 3 of ref 17. For training the DDNN methods, we have used a regular grid of 100 points in the *R*_S–H_ range for which the adiabatic energies have been calculated while the elements of the DPEM have only been given at the boundary points of the *R*_S–H_ interval. To get a better idea, adiabatic energies and the model DPEM are plotted in Figure 10.

**Figure 10:**
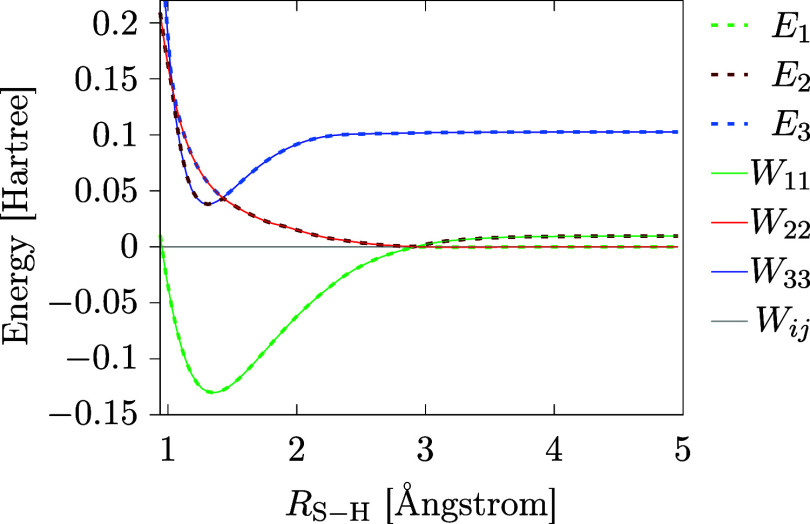
Adiabatic energies (dashed lines) and elements of the DPEM (solid lines) for the three state model of thiophenol as a functions of *R*_S–H_ (S–H bond distance) as obtained from ref 29.

In analogy with the numerical experiments performed for model $M_{3}$ , we have run a total of 3072 independent trainings of the DDNN_G_^*n*^ (*n* = 1 – 5) with the regularization term weight set to α_r_ = 10^–8^. For comparison, α_r_ = 10^–4^ has also been considered for *n* = 1 (as used in ref 17). Median and minimum values of $D^{2}$ are presented in Table 7, corresponding cumulative distribution functions of $D^{2}$ are plotted in Figure 11.

**Figure 11:**
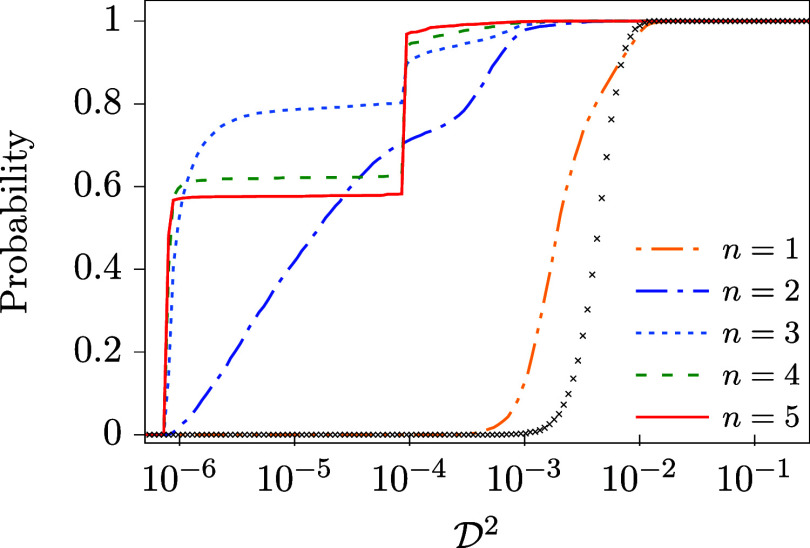
Cumulative distribution functions of $D^{2}$ calculated for DDNN_G_^*n*^ (*n* = 1–5). Distribution function plotted as black crosses corresponds to the setting of the DDNN method as it was used in ref 17, i.e., a linear activation function in the DPEM layer and α_r_ = 10^–4^.

**Table 7:** Median and Minimum Values of $D^{2}$ Calculated for Different Values of *n* in the DDNN_G_^*n*^ Method^a^

DDNN_G_^*n*^	med $\left(D^{2}\right)$	min $\left(D^{2}\right)$
*n* = 1	4.38·10^–3^	4.68·10^–4^
*n* = 1	1.93·10^–3^	2.32·10^–4^
*n* = 2	1.61·10^–5^	7.95·10^–7^
*n* = 3	9.62·10^–7^	7.54·10^–7^
*n* = 4	8.15·10^–7^	7.51·10^–7^
*n* = 5	8.04·10^–7^	7.52·10^–7^

a All the data have been obtained from 3072 independent ANN trainings. In the first line (typed in italics), values obtained for the setting used in ref 17 (α_r_ = 10^–4^) are shown for comparison.

Like for models $M_{2}$ and $M_{3}$ , we can observe, both in Table 7 and in Figure 11, that increasing the value of *n* above *n* = 1 leads to a better performance of the method. However, for *n* ≥ 3, the behavior of the method does not change significantly. Even though the value of med $\left(D^{2}\right)$ decreases slightly with increasing *n* in this range, from individual distribution functions we may conclude that it is preferable to use *n* = 3. Moreover, comparing the distribution functions with the result corresponding to the original setting of the DDNN method as used in ref 17, we may conclude that using α_r_ = 10^–8^ instead of α_r_ = 10^–4^ leads to a well visible improvement for this system, like for models $M_{2}$ and $M_{3}$ . To further illustrate the performance of the DDNN_G_^*n*^ methods achieved for different settings, the best results obtained with respect to the value of $D^{2}$ are shown in Figure 12.

**Figure 12:**
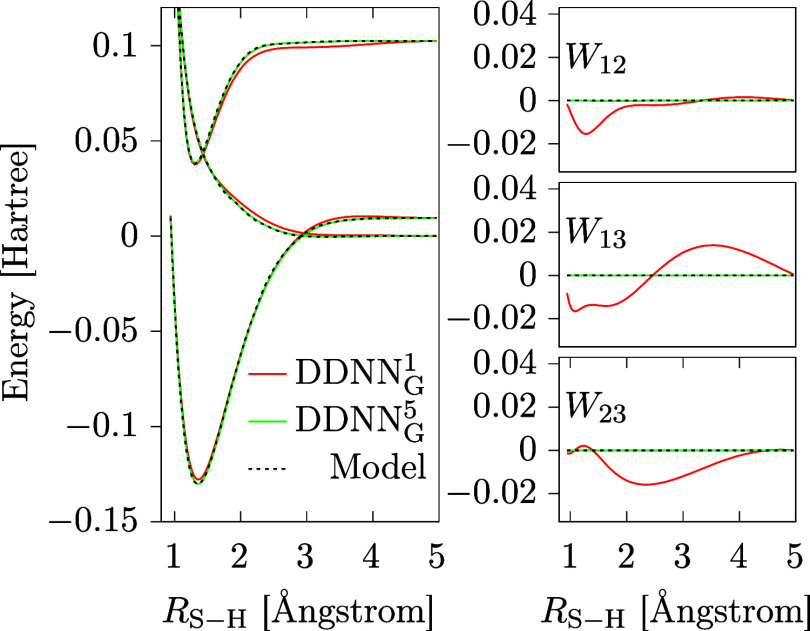
Best results with respect to the value of $D^{2}$ as obtained using the DDNN_G_^1^ and DDNN_G_^5^ methods. The best DDNN approximations are plotted as solid colored lines, benchmark values taken from ref 29 are shown, for comparison, as dotted black lines.

Finally, we have found that employing the DTSG algorithm (Section 3.3) does not lead to any significant improvement. Particularly for *n* ≥ 3, the DDNN_G_^*n*^ methods give accurate results with a high probability and increasing the number of diabatic training points only leads to a marginal improvement at the cost of a much longer computational time needed for the method training. We have also considered the PMAF introduced in ref 19 to improve the asymptotic behavior of the DDNN approximation. A few results of selected tests performed for various settings of PMAF internal parameters are provided in Section 4 of the Supporting Information Material.

#### 3.4.2 Thiophenol: Example 2

For completeness, we have also tested the DDNN_G_^*n*^ method for a system which is denoted as Example 2 in ref 17, namely the Nonintuitive diabatic crossing in thiophenol, consisting of potential energy curves of thiophenol corresponding to a 1D cut along the C–C–S–H torsional angle, ϕ, with the S–H bond length, *R*_S–H_, fixed at 1.35 Å, and all the remaining atoms kept at their equilibrium positions (see ref 29 for details). Adiabatic energies and the target DPEM are plotted for this example in Figure 13.

**Figure 13:**
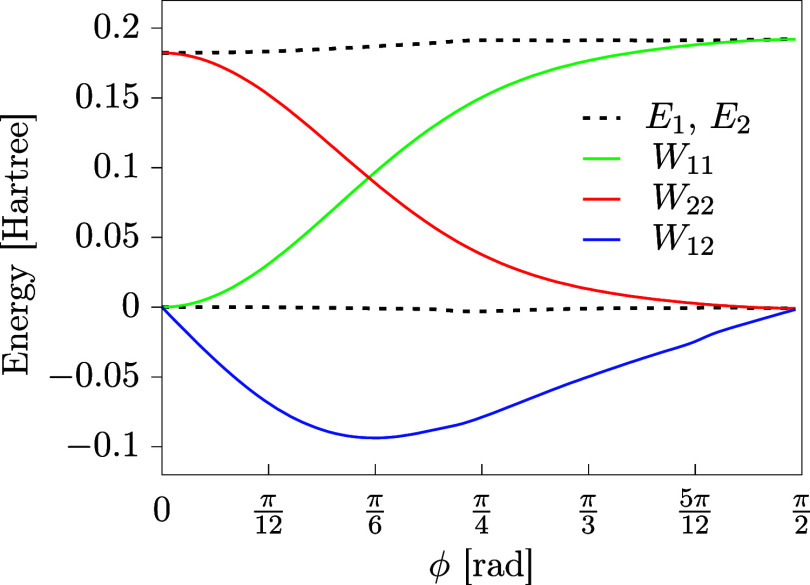
Two state model of thiophenol energy as a function of the C–C–S–H torsional angle (ϕ). Elements of the DPEM are plotted as solid lines and adiabatic energies are given as dashed lines. All the energies have been taken from ref 29.

As in example 1, the DDNN_G_^*n*^ methods (*n* = 1–5) have been used to represent the diabatic curves, and since the present example comprises only two states, 4736 independent trainings of the DDNN_G_^*n*^ methods have been performed for each *n*. Resulting median and minimum values of $D^{2}$ are presented in Table 8, corresponding cumulative distribution functions of $D^{2}$ are shown in Figure 14.

**Figure 14:**
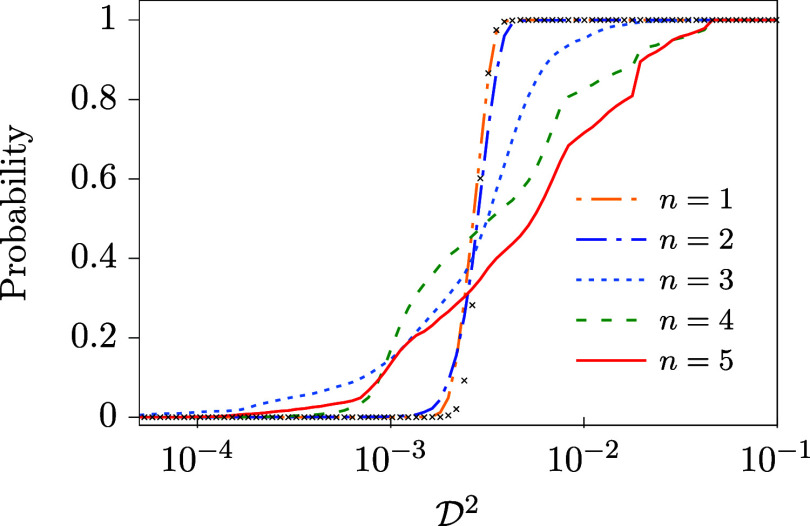
Cumulative distribution functions of $D^{2}$ calculated using the DDNN_G_^*n*^ method (*n* = 1–5). The distribution function plotted as black crosses corresponds to the setting of the DDNN method used in ref 17, i.e., linear activation fuction in the DPEM layer and α_r_ = 10^–4^.

**Table 8:** Median and Minimum Values of $D^{2}$ for Different Values of *n* in DDNN_G_^*n*^ ^a^

DDNN_G_^*n*^	med $\left(D^{2}\right)$	min $\left(D^{2}\right)$
*n* = 1	2.82·10^–3^	1.46·10^–3^
*n* = 1	2.67·10^–3^	1.28·10^–3^
*n* = 2	2.81·10^–3^	1.61·10^–4^
*n* = 3	3.17·10^–3^	2.33·10^–5^
*n* = 4	3.26·10^–3^	1.26·10^–4^
*n* = 5	5.43·10^–3^	3.48·10^–5^

a All the data have been obtained from 4736 independent ANN trainings. Like in Table 7, the first line (typed in italics) corresponds to the setting used in ref 17 (α_r_ = 10^–4^).

Interestingly, as is seen from Table 8, the values of med $\left(D^{2}\right)$ are comparable to one another for all the considered values of *n*; more specifically, they slightly grow up with increasing *n*. For min $\left(D^{2}\right)$ , on the other hand, a significant improvement of the DDNN_G_^*n*^ performance is seen for higher values of *n*. It is further clear from the corresponding distribution functions (see Figure 14) that the range of $D^{2}$ is much broader for higher values of *n* (*n* ≥ 3) even though corresponding values of med $\left(D^{2}\right)$ are close to each other. It highly likely means that, on one hand, increasing *n* improves the adaptability of the ANN approximation and leads thus to considerably improved best solutions, but at the same time contaminates the outputs of the ANN trainings with a non-negligible amount of inferior solutions, which are probably not accessible at lower values of *n*. To show this more explicitly, in Figure 15 we compare the best solutions obtained for the DDNN_G_^1^ (*n* = 1) and DDNN_G_^3^ (*n* = 3) methods, representing respectively the worst and the best performance of the DDNN_G_^*n*^ methods in the case of this particular example.

**Figure 15:**
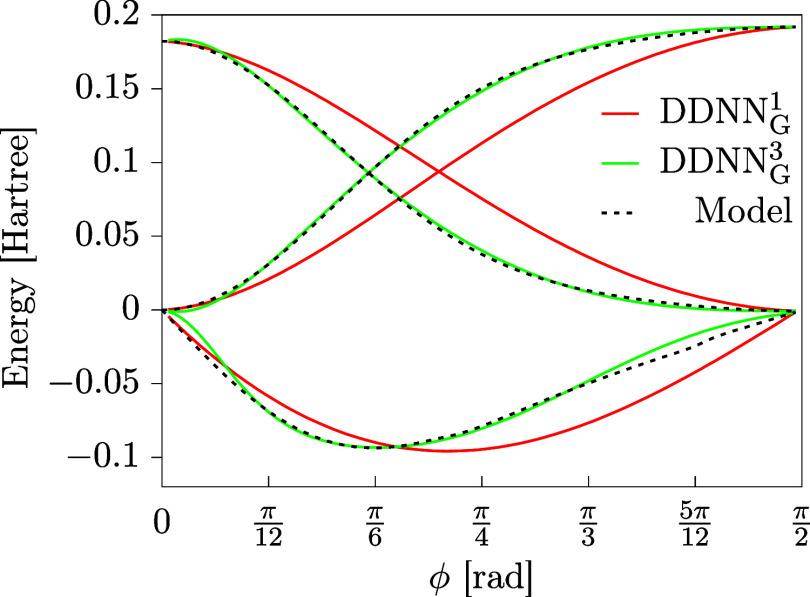
Best results with respect to the value of $D^{2}$ as obtained using the DDNN_G_^1^ and DDNN_G_^3^ methods. The ANN approximations are plotted as solid colored lines, the benchmark values taken from ref 29 are shown, for comparison, as dotted black lines.

## 4 Conclusions

A recently proposed approach to diabatization through artificial neural networks (ANNs)^17^ has been tested thoroughly against two artifical 1D models comprising two and three electronic states with one or two avoided crossings, respectively, and a more realistic example of 1D cuts through the thiophenol potential energy surface. The approach consists in proposing an ANN with two output layers, one for the diabatic potential energy matrix (DPEM) and the other for adiabatic energies, interconnected through DPEM diagonalization. Most importantly, training sets used to optimize the ANN contain prevailingly adiabatic energies with the full DPEM added only at points where the adiabatic and diabatic represenations are (at least approximately) identical. As a consequence, no additional calculations to those of adiabatic energies are needed.

In this work, we have provided an analysis of performance of the ANN diabatization approach introduced in ref 17 and proposed several modifications that visibly improve its performance. In particular, we have studied the effect (and optimal choice) of the activation functions used in ANN hidden layers and in the DPEM layer, the role of the regularization term added to the loss function used for ANNs training, and the role of the structure of training sets. To provide a global (statistical) view of the performance of the proposed diabatization approach, repeated trainings have been performed for each particular setting and statistical parameters like the median and mean values of mean-square-error deviations of trained ANNs from a particular model and the probability of getting a sufficiently accurate ANN representation of the DPEM in a single training event have been calculated.

The main lessons which can be derived from the performed analysis may be summarized as follows. One, three activation functions have been tested for hidden layers of the ANNs, the sigmoid function, the hyperbolic tangent, and the Gaussian Error Linear Unit (GELU) function.^27^ More or less a balanced performance have been seen for all the three functions with GELU slightly overperfoming the two others. Two, the identity activation function, *g*(*z*) = *z* is usually used for the DPEM layer in the literature. Here, we tested several power functions, *g*(*z*) = *z* ^*k*^, *k* ≤ 5, and have found that using higher powers than *k* = 1 significantly improves the performance of the ANN diabatization approach. Three, the role of the Tikhonov regularization term^22^ has been confirmed for the two-state model, but quite surprisingly, almost no positive effect of the regularization term has been observed for the (more complex) three-state model. It seems to indicate that the role of the regularization term in the specific problems discussed in this work is not unambiguous and that a more detailed analysis would be useful. Four, the original method of ref 17 proposes (for 1D models) to include only (two) points with the full DPEM provided, namely those at the lower and upper limit of the nuclear coordinate interval where the adiabatic and diabatic representations are presumed to coincide. In this way, any time-consuming calculation of the DPEM can be avoided. While it has proved fully sufficient for the simpler two-state model analyzed in this work, in the case of the more involved three-state model such a restriction imposed on the training set does not yield sufficiently accurate solutions. As a consequence, we have extended the number of diabatic training points by a specific algorithm described in Section 3.3. A significant improvement (by an order of magnitude) has been achieved by using this algorithm. Finally, we have presented results of similar tests of the DDNN method performed for two more realistic models represented by two different 1D cuts of a thiophenol potential energy surface. The conclusions obtained for the two preceding artificial models have been fully confirmed.

In conclusion, when testing the reliability of the DDNN method based on the use of ANNs, we have found that after performing a sufficient number of trainings, some reasonably (or even highly) accurate solutions are found. However, what is also clear from the distributions of errors presented in this work is that even for very simple 1D models, the probability of finding a correct diabatic representation may be considerably limited unless the method is well tuned. It means that the method itself is very sensitive to the values of the internal parameters and the appropriate setting of them can significantly improve its performance.

Despite quite a detailed analysis of the diabatization approach of ref 17 has been performed for selected 1D models, a lot of questions remain unsolved. For example, neither many-state models with a complex structure of interacting electronic states have been considered nor models comprising more than one nuclear coordinate. Further, the models analyzed in this work include diagonal elements of the DPEM which are linear functions of the coordinate variable. The effect of nonlinearities in these terms on the accuracy of trained ANNs has not been investigated systematically (though preliminary calculations we performed seem to indicate that it may be significant). Most importantly, however, while we have shown that the proposed approach is able to find very accurate ANN representations of tested 1D models, it is not clear how to pick up the best one from many provided by repetitive trainings without the need of time-consuming calculations of extended validation sets comprising a lot of points with the full DPEM provided. The latter issue is currently being analyzed and will be the subject of a future work.

## Supplementary Material



## Footnotes

1 Mathematically, the task solved by the method proposed in ref 17 means that one looks for a DPEM in a region of nuclear coordinates the eigenvalues of which are known ”everywhere” while its eigenvectors are known only on the boundary of the region. Even if a smooth dependence on nuclear coordinates is required for both, the task has infinitely many solutions.

2 The criterion we introduce here may serve as a quantitative counterpart of a frequently used qualitative statement that “a graphical comparison shows a good agreement”, namely because the range on the vertical axis in a typical graphical representation of obtained results is usually comparable to Δ_*j*_.

3 The minima of *d*_*j*_(*x*) detect avoided crossings of considered adiabatic states, i.e., locate points close to which corresponding diabatic states are expected to cross.

## Notes

Additional explanations and data pertinent to the present study and not included therein to preserve brevity (PDF)
