# Supplementary material for: Tests of Artificial Neural Network-Based Diabatization Approaches on Simple 1D Models
Source: J Chem Theory Comput. 2025 Jul 15;21(15):7199–211. doi: 10.1021/acs.jctc.5c00083 (PMC12355706; doi:10.1021/acs.jctc.5c00083)
Supplement: Supplementary file 1 [file ct5c00083_si_001.pdf]

Supporting information to "Tests of artificial neural network based diabatization approaches on simple 1D models"

Martina Ćosićová,[*,†,‡] Thierry Leininger,[‡] and René Kalus[†]

†*Department of Applied Mathematics, VSB – Technical University of Ostrava, 17. listopadu 2172/15, 70800 Ostrava, Czech Republic*

‡*Laboratoire de Chimie et Physique Quantiques, Université de Toulouse, CNRS UMR5626, 118 route de Narbonne, 31062 Toulouse Cedex 09, France*

E-mail: martina.cosicova@vsb.cz

Abstract

In this supplementary information file, some auxiliary explanations and data sets which are pertinent to the material presented in the paper and not included therein for brevity are given for readers' convenience.

1 List of symbols

Used symbol		Description
This work	Ref. 1 (Ref. 17 in the paper)	
N_{S}	N	number of states
$\mathbf{W}$	$\mathbf{U}$	DPEM
W_{ij}	$\mathbf{U}_i$	elements of DPEM
$\mathbf{E}$	$\mathbf{V}$	vector of adiabatic energies
E_i	$\mathbf{V}_i$	i-th adiabatic energy
N_{H}		number of hidden layers in neural network
K_i		number of neurons in i-th hidden layer
N_{D}		number of neurons in DPEM layer
$y_k^{(j)}$	$\mathbf{a}_k^j$	k-th neuron of j-th hidden layer
$\mathbf{y}^{\mathrm{D}}$		DPEM layer
y_k^{D}	$\mathbf{a}_k^{L-1}$	k-th neuron of DPEM layer
$\mathbf{y}^{\mathrm{A}}$		adiabatic layer
y_k^{A}	$\mathbf{a}_k^L$	k-th neuron of adiabatic layer
N_{TD}	n_C	number of diabatic training points
N_{TA}	n	number of adiabatic training points
x_i^{D}		i-th diabatic training point
x_i^{A}		i-th adiabatic training point
α_{r}	α_2	weight of regularization term of loss function

2 Adiabatic and diabatic representations

In this section, the basics of adiabatic and diabatic representations are provided for reader's convenience. Since it is not the main topic of the present paper, just a brief summary relevant to its scope is included and the reader is referred to the cited literature (see, e.g., Refs. 2

and 3) for more details.

Let us consider a system consisting of heavy (slow) and light (fast) particles, e.g., atomic nuclei and electrons, respectively. At the quantum level, the state of this system is described by a multidimensional wave function, $\Psi(\mathbf{r}, \mathbf{R}, t)$, where $\mathbf{r}$ and $\mathbf{R}$ denote collective coordinates of the fast and slow particles, respectively, and t is a time variable. The dynamics of the system is then governed by a time-dependent Schrödinger equation,

$$\hat{\mathcal{H}}\Psi = i\hbar\frac{\partial\Psi}{\partial t}, \tag{1}$$

where $\hat{\mathcal{H}}$ is the overall Hamilton operator of the system and $\hbar$ denotes the reduced Planck constant. In the following, we will use the atomic units and will set $\hbar = 1$.

The Hamilton operator is often written in a separated form, $\hat{\mathcal{H}} = \hat{\mathcal{T}} + \hat{\mathcal{W}}$, where

$$\hat{\mathcal{T}} = -\frac{1}{2}\Delta_{\mathrm{R}} \tag{2}$$

is the operator of the kinetic energy of slow particles with Δ_{R} being a multidimensional Laplace operator with respect to mass-weighted[2] slow-particles coordinates, and

$$\hat{\mathcal{W}} = -\frac{1}{2}\Delta_{\mathbf{r}} + V(\mathbf{r}, \mathbf{R}) \tag{3}$$

is the so called fast-particles (electronic) Hamiltonian. Like for the slow particles, $\Delta_{\mathbf{r}}$ denotes a multidimensional Laplace operator with respect to fast-particles coordinates, and $V(\mathbf{r}, \mathbf{R})$ represents an overall interaction potential of the system.

To make Eq. (1) numerically tractable, an expansion of the total wave function against a set of eigenfunctions of $\hat{\mathcal{W}}$ is often used,

$$\Psi(\mathbf{r}, \mathbf{R}, t) = \sum_j \chi_j(\mathbf{R}, t)\,\psi_j(\mathbf{r}, \mathbf{R}), \tag{4}$$

where

$$\hat{\mathcal{W}}\psi_j(\mathbf{r},\mathbf{R}) = E_j(\mathbf{R})\,\psi_j(\mathbf{r},\mathbf{R}). \tag{5}$$

In general, the sum on the right-hand side of Eq. (4) should be infinite, in practice however, a finite number of fast-particles states (wave functions) is used. Only the fast-particles states relevant for the particular problem are considered and the others are ignored.

If the expansion of Eq. (4) is inserted, together with definition Eqs. (2) and (3), into the Schrödinger equation, Eq. (1), one gets, after some algebra, the following set of equations for unknown functions $\chi_j(\mathbf{R},t)$,

$$-\frac{1}{2}\Delta_{\mathbf{R}}\chi_j + E_j\chi_j + \sum_k \left[G_{jk} - \mathbf{F}_{jk}\cdot\nabla_{\mathbf{R}}\right]\chi_k = i\hbar\frac{\partial\chi_j}{\partial t}, \tag{6}$$

where $\mathbf{F}_{jk} \equiv \langle\psi_j|\nabla_{\mathrm{R}}\psi_k\rangle$ (with ∇_{R} being a multidimensional gradient operator with respect to the slow-particles coordinates) and $G_{jk} \equiv \frac{1}{2}\langle\psi_j|\Delta_{\mathrm{R}}\psi_K\rangle$. Eqs. (6) give the *adiabatic representation* of the dynamics of the system under study and the corresponding fast-particles basis set wave functions, ψ_j, are usually denoted as *adiabatic wave functions.*

Eqs. (6) are only of a limited use in numerical simulations, however, since divergences of the $\mathbf{F}_{jk}$ terms occur in regions of $\mathbf{R}$ where fast-particles states cross. For this reason, different representations are often used,

$$\Psi(\mathbf{r},\mathbf{R},t) = \sum_j \omega_j(\mathbf{R},t)\,\phi_j(\mathbf{r},\mathbf{R}), \tag{7}$$

within which the diverging terms are removed. These representations are collectively denoted as *diabatic representations* and the corresponding fast-particles basis sets, ϕ_j, as *diabatic basis sets.* We use the plural in the preceding sentence since the diabatic basis sets are not unique.[4] Moreover, they do not exist in a strict sense if a finite number of diabatic fast-particles wave functions is considered. In fact, we should speak about *approximately* diabatic basis sets for which the non-adibatic couplings are non-zero but negligible (as small as possible).[5] See also

Ref. 3 for a more complete discussion.

It can be shown[6] that an approximately diabatic basis set can be obtained by an orthogonal transformation of the adiabatic basis set and that the unknown diabatic expansion coefficients, ω_j, can be obtained by solving a set of equations reading

$$-\frac{1}{2}\Delta_{\mathbf{R}}\omega_j + \sum_k W_{jk}\omega_k = i\hbar\frac{\partial \omega_j}{\partial t}, \tag{8}$$

where $W_{jk} = W_{jk}(\mathbf{R}) \equiv \langle \phi_j \,|\, \hat{\mathcal{W}} \,|\, \phi_k \rangle$ are elements of a *diabatic potential energy matrix* (DPEM). Noteworthy, DPEMs are in principle free of any divergences and depend smoothly on $\mathbf{R}$. The adiabatic representation is obtained from the diabatic one by diagonalizing the corresponding DPEM. The eigenvalues of the DPEM give the adiabatic energies, E_j, and the DPEM eigenvectors define the diabatic-to-adiabatic transition.

3 The *diabatic training set generating* (DTSG) algorithm

In the following text, we describe in detail the algorithm for generating additional diabatic training points which has been introduced in Sec. 3.3 of the paper. Before we discuss the algorithm itself, a few preliminary definitions are needed.

Firstly, for $i = 1, \cdots, N_{\mathrm{S}}$ (N_{S} is the number of electronic states considered), we define a set of functions quantifying vertical distances between relevant adiabatic states,

$$d_i(x) = E_{i+1}(x) - E_i(x), \tag{9}$$

where E_j denotes j-th adiabatic energy (sorted in ascending order), and presume the following conditions to be valid:

- adiabatic and diabatic energies (diagonal elements of the DPEM) are close to each

other outside the regions of avoided crossings so that they can be considered equal (for simplicity, we also presume that the avoiding crossings do not take place at the end points of the interval of the x parameter),

- the diabatic energy curves cross in and/or close to the minima of functions $d_i(x)$.

Secondly, a permutation, π_L, is introduced which maps, at the left-most point of the considered interval of x, $I = [x_\mathrm{L}, x_\mathrm{R}]$, the diabatic energies to the adiabatic ones,

$$\begin{pmatrix} E_1(x_\mathrm{L}) \\ \vdots \\ E_{N_\mathrm{S}}(x_\mathrm{L}) \end{pmatrix} = \pi_\mathrm{L} \begin{pmatrix} W_{11}(x_\mathrm{L}) \\ \vdots \\ W_{N_\mathrm{S}N_\mathrm{S}}(x_\mathrm{L}) \end{pmatrix}. \tag{10}$$

Thirdly, let us further define an auxiliary vector,

$$\mathbf{v}_\mathrm{L} = \pi_\mathrm{L} \begin{pmatrix} 1 \\ \vdots \\ N_\mathrm{S} \end{pmatrix}, \tag{11}$$

which represents the ordering of the diabatic states at x_L according to the values of their energies.

The workflow of the algorithm can then be summarized as follows.

Firstly, all the minima of functions d_i are found and represented by couples $\{x_k, i(k)\}$, $k = 1, \ldots M$, where x_k denotes the position of k-th minimum (we assume x_k sorted in an ascending order) and $i(k)$ is the index of function d developing the minimum at x_k.

Secondly, a sequence of vectors $\mathbf{v}_\mathrm{L} \equiv \mathbf{v}_0, \mathbf{v}_1, \mathbf{v}_2, \cdots, \mathbf{v}_M$ is constructed recursively,

$$\mathbf{v}_k = \rho_{i(k)} \mathbf{v}_{k-1}, \tag{12}$$

with $\rho_{i(k)}$ being a transposition operator of the $i(k)$-th and $(i(k)+1)$-th elements of the vector standing to the right. Vector $\mathbf{v}_k$ provides the order of diabatic states between points

x_k and x_{k+1} and can be written, if one denotes $\pi_k = \rho_{i(k)} \circ \rho_{i(k-1)} \circ \cdots \circ \rho_{i(1)}(\mathbf{v}_\mathrm{L})$ where the circus represents the composition of transpositions, as

$$\mathbf{v}_k = \pi_k \mathbf{v}_\mathrm{L}. \tag{13}$$

Thirdly, a system of subsets of interval I is constructed,

$$\mathcal{S}_i = I \setminus \left(\bigcup_{a_k = i} B(x_k; r_1) \cup \bigcup_{b_k = i} B(x_k; r_1) \right), \tag{14}$$

where $i = 1, \ldots, N_\mathrm{S}$, $B(x; r) = [x - r; x + r]$, $a_k = (\mathbf{v}_k)_{i(k)}$, and $b_k = (\mathbf{v}_k)_{i(k)+1}$, and

$$\mathcal{S}_{lm} = I \setminus \bigcup_{a_k = l, b_k = m} B(x_k; r_2), \tag{15}$$

with $l = 1, \ldots, N_\mathrm{S} - 1$ and $m = l + 1, \ldots, N_\mathrm{S}$. Parameters $r_1 > 0$ and $r_2 > 0$ represent radii of regions which are assumed to be "close" to the detected minima for the diagonal and off-diagonal elements of the DPEM, respectively, and are to be set appropriately by the user.

Since the values of x_k, $a_k = (\mathbf{v}_k)_{i(k)}$, and $b_k = (\mathbf{v}_k)_{i(k)+1}$ obtained in step two specify that there is an avoided crossing between states $E_{i(k)}$ and $E_{i(k)+1}$, and therefore diabatic states W_{a_k} and W_{b_k} cross at x_k, the $\mathcal{S}_i$ sets give the regions of x where corresponding diabatic and adiabatic curves coincide and the $\mathcal{S}_{lm}$ sets represent regions where the off-diagonal elements of the DPEM may be approximated by zero. Naturally then, the last step of the algorithm consists of the following: a) for $x \in \mathcal{S}_{lm}$, additional points are added for off-diagonal elements of the DPEM with zero values,

$$W_{lm}(x) = 0, \tag{16}$$

and the diagonal part of the DPEM is set to corresponding adiabatic energies,

$$W_{ii}(x) = \left(\pi_\mathrm{L}^{-1} \circ \pi_k^{-1} \mathbf{E}(x) \right)_i \tag{17}$$

for a grid of points selected from $x \in [x_k; x_{k+1}] \cap \mathcal{S}_i$.

An illustration of an extended diabatic training set resulting from the present algorithm is provided for model $\mathbb{M}_3$ (see Sec. 2.2 of the paper) and for a specific choice of r_1 and r_2 in Figure 8 of Sec. 3.3 of the main paper.

4 An example of the use of parametrically-managed post-activation function

In this section, we present a few results of testing an approach introduced in Ref. 7, where authors define parametric post-activation function to calculate the values of individual elements, y_k^{D}, in DPEM output layer as follows,

$$y_k^{\mathrm{D}}\left(z_k^{\mathrm{D}}; R\right) = z_k^{\mathrm{D}} \cdot f_1(R) \cdot f_2(R), \tag{18}$$

with

$$f_1(R) = \frac{1}{2} + \frac{1}{2}\tanh\left(f(R-a)\right),$$
$$f_2(R) = \frac{1}{2} + \frac{1}{2}\tanh\left(f(-R+b)\right),$$

being functions of inter-nuclear distances, R, depending on three real parameters, f, a, and b. For simplicity, we shall denote $g = f_1 \cdot f_2$ and refer to this function as "post-activation" function. Thanks to its specific shape, this function is mainly used to dampen the energy value in specific areas.

In this section, we test the application of the post-activation function on the one-dimensional cut of thiophenol energy depending on the S-H distance, $R_{\mathrm{S-H}}$, i.e., the example described in Section 3.4 of the main text. Taking into account the values of diagonal elements of DPEM for large $R_{\mathrm{S-H}}$, we see that it cannot be advantageous to multiply them by damping function.

Therefore, in the following numerical experiment, the DPEM output values are defined by

$$y_k^{\mathrm{D}} = \begin{cases} z_k^{\mathrm{D}}, & 1 \le k \le N_{\mathrm{S}} \\ \\ g_{\mathrm{D}}\left(z_k^{\mathrm{D}}\right) \cdot g(R), & N_{\mathrm{S}} < k \le N_{\mathrm{D}} \end{cases} \tag{19}$$

with g_{D} being an AF used in the DPEM layer to represent the off-diagonal elements, and $g = f_1 f_2$ is the post-activation function. To test the significance of the function g, we have defined two sets of its parameters, creating two variants, g_1 and g_2. Individual settings are given by Table 1. In Figure 1, we have plotted both g_1 and g_2.

Table 1: Values of internal parameters, f, a, and b defining the post-activation functions g_i.

	f	a	b
g_1	2.0	-2.0	4.0
g_2	2.0	2.0	4.0

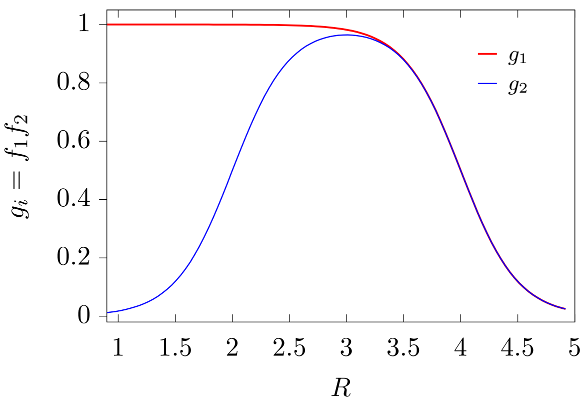

Figure 1: Post-activation functions g_i given by parameters in Table 1.

To test the influence of using different damping functions g, we have performed a total of 750 independent trainings using $\mathrm{DDNN}_{\mathrm{G}}^1$, and $\mathrm{DDNN}_{\mathrm{G}}^3$, started from different initial guesses of the weights and biases generated by the Glorot method. Median and minimum values of $\mathcal{D}^2$ are presented in Table 2. In Figure 2, we have plotted corresponding cumulative distribution functions of $\mathcal{D}^2$.

Table 2: Median and minimum values of $\mathcal{D}^2$ for different values of $\mathrm{DDNN}_\mathrm{G}^1$ and $\mathrm{DDNN}_\mathrm{G}^3$ for different post-activation functions, g. All the data have been obtained from 750 independent ANN trainings.

	med($\mathcal{D}^2$)	min($\mathcal{D}^2$)
$\mathrm{DDNN}_\mathrm{G}^1$		
$g = 1$	$1.79{\cdot}10^{-3}$	$3.12{\cdot}10^{-4}$
$g = g_1$	$1.28{\cdot}10^{-3}$	$8.65{\cdot}10^{-8}$
$g = g_2$	$7.57{\cdot}10^{-4}$	$5.46{\cdot}10^{-8}$
$\mathrm{DDNN}_\mathrm{G}^3$		
$g = 1$	$9.49{\cdot}10^{-7}$	$7.66{\cdot}10^{-7}$
$g = g_1$	$3.39{\cdot}10^{-7}$	$5.69{\cdot}10^{-8}$
$g = g_2$	$2.23{\cdot}10^{-6}$	$5.97{\cdot}10^{-8}$

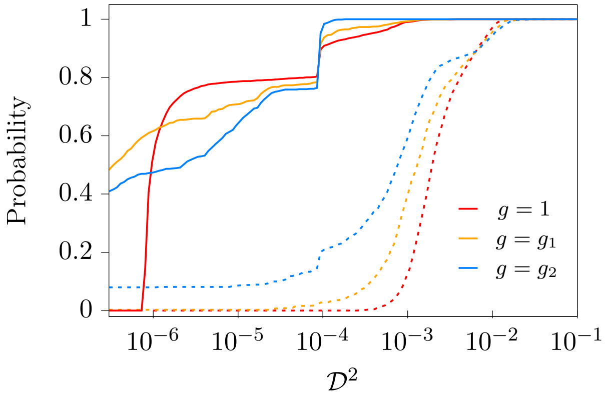

Figure 2: Cumulative distribution functions of $\mathcal{D}^2$ calculated for $\mathrm{DNN}_\mathrm{G}^1$ (dotted line), and $\mathrm{DNN}_\mathrm{G}^3$ (solid line) for different post-activation functions defined by Table 1. Red color correspond to the results without the use of any damping function.

It is clear from the obtained results that the use of the parametric post-activation function significantly affects the behavior of the diabatization method. In particular, we see the biggest changes regarding the results with a high degree of accuracy, i.e., whose error $\mathcal{D}^2$ is below the value of 10^{-6}, which can be seen both in the last column of Table 2, and Figure 2. We naturally observe more significant changes with method $\mathrm{DDNN}_\mathrm{G}^1$, which by itself showed a much worse success rate.

The success of using the post-activation function is largely due to the fact that we know in advance the desired character of some elements of the DPEM in certain regions. In case of this example, we know that the off-diagonal terms of the DPEM have almost zero values over

the entire studied interval. It can therefore be expected that the use of a damping function, which has the task of reducing the value to zero, is very convenient. Otherwise, in case we do not have this type information, using the post-activation function can be problematic because we may not know how to set its internal parameters.

References

(1) Shu, Y.; Truhlar, D. G. Diabatization by Machine Intelligence. *Journal of Chemical Theory and Computation* **2020**, *16*, 6456–6464.

(2) Tully, J. C. In *Modern Methods for Multidimensional Dynamics Computations in Chemistry*; Thompson, D. L., Ed.; World Scientific: Singapore, 1998; pp 34–72.

(3) Shu, Y.; Varga, Z.; Kanchanakungwankul, S.; Zhang, L.; Truhlar, D. G. Diabatic States of Molecules. *The Journal of Physical Chemistry A* **2022**, *126*, 992–1018.

(4) Mead, C. A.; Truhlar, D. G. Conditions for the Definition of a Strictly Diabatic Electronic Basis for Molecular Systems. *The Journal of Chemical Physics* **1982**, *77*, 6090–6098.

(5) Kendrick, B. K.; Mead, C. A.; Truhlar, D. G. Properties of Nonadiabatic Couplings and the Generalized Born-Oppenheimer Approximation. *Chemical Physics* **2002**, *277*, 31–41.

(6) Baer, M. Adiabatic and diabatic representations of atom-molecule collisions: Treatment of the collinear arrangement. *Chemical Physics Letters* **1975**, *35*, 112–118.

(7) Akher, F. B.; Shu, Y.; Varga, Z.; Bhaumik, S.; Truhlar, D. G. Post-Activation Function for Managing the Behavior of a Neural Network Potential with a Low-Dimensional Potential. **2023**,
